# Supplementary material for: Non-Antibiotic Antimony-Based Antimicrobials
Source: Molecules. 2022 Oct 23;27(21):7171. doi: 10.3390/molecules27217171 (PMC9654735; doi:10.3390/molecules27217171)
Supplement: Supplementary file 1 [file molecules-27-07171-s001.zip › molecules-1950607-supplementary.pdf]

Pages of Supporting Information for the paper

Non-antibiotic antimony-based antimicrobials.

by

Nikolay Gerasimchuk[1*], Kevin Pinks[1], Tarosha Salpadoru[2], Kaitlyn Cotton[2], Olga Michka[2], Marianna Patrauchan[2*] and Karen L. Wozniak[2]

Actual photographs under microscope of crystalline thallium(I) cyanoximates containing thioamide groups: **A** – Tl(TCO), **B** - Tl(TDCO) and **C** - bulk powders of these compounds. Elemental analysis data for the latter compound are in the lower panel.

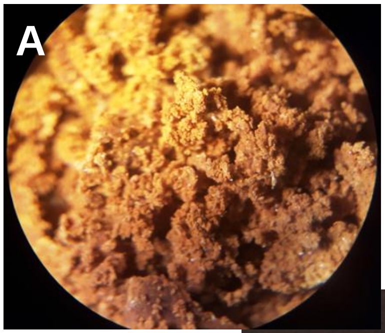

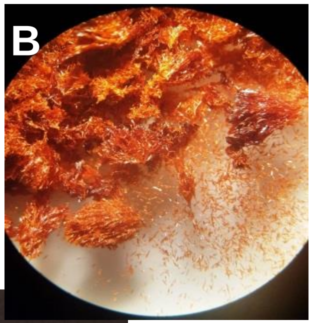

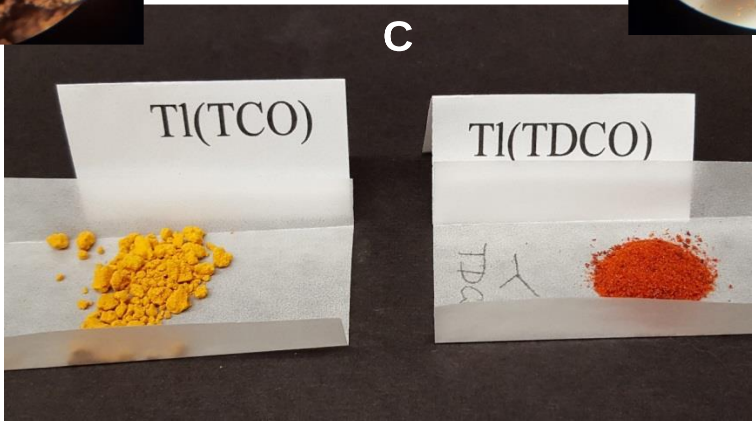

ATLANTIC MICROLAB, INC.

Sample No. Tl(TDCO)-batch-1

6180 Atlantic Blvd. Suite M
Norcross, GA 30071

www.atlanticmicrolab.com

PROFESSOR/SUPERVISOR: Prof. N. Gerasimchuk

PO# / CC#:

SUBMITTER

Company / School Missouri State University

Address 901 South National Avenue, Temple Hall 456

Dept. Chemistry

NAME Kevin Pinks DATE 12/21/18

PHONE (417) 836-55-06

Element	Theory	Found	
C	16.6600	16.84	
H	1.6800	1.61	
N	11.6500	11.77	

Single ☑ Duplicate ☐

Elements Present:

Analyze for:

Hygroscopic ☐ Explosive ☐

M.P. B.P.

To be dried: Yes ☐ No ☐

Temp. Vac. Time

RUSH SERVICE ☐ Rush service guarantees analyses will be completed and results available by 5pm EST on the day the sample is received by 11am.

Include Email Address or Fax # Below

Date Received DEC 27 2018 Date Completed DEC 28 2018

Remarks:

Instrumentation and accessories used for separation of organoantimony(V) cyanoximates from Ag(I)Br. A Thermo Scientific centrifuge (left) with nylon Eppendorf tubes and an insert for filtering (right, with arrow).

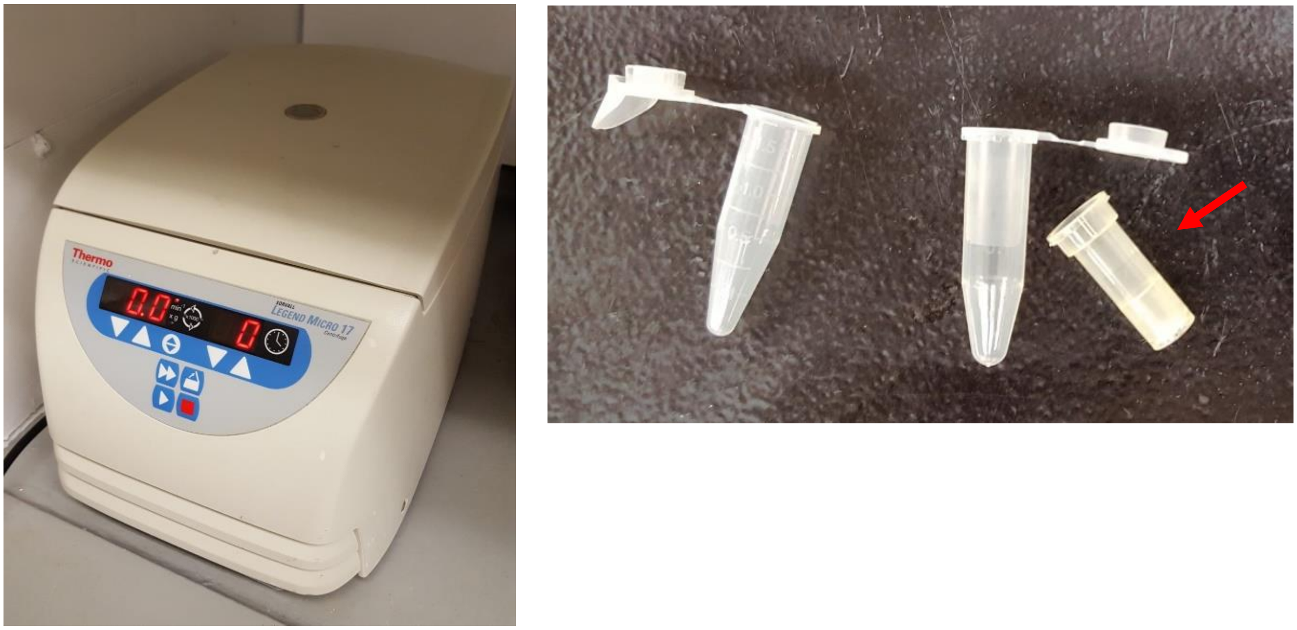

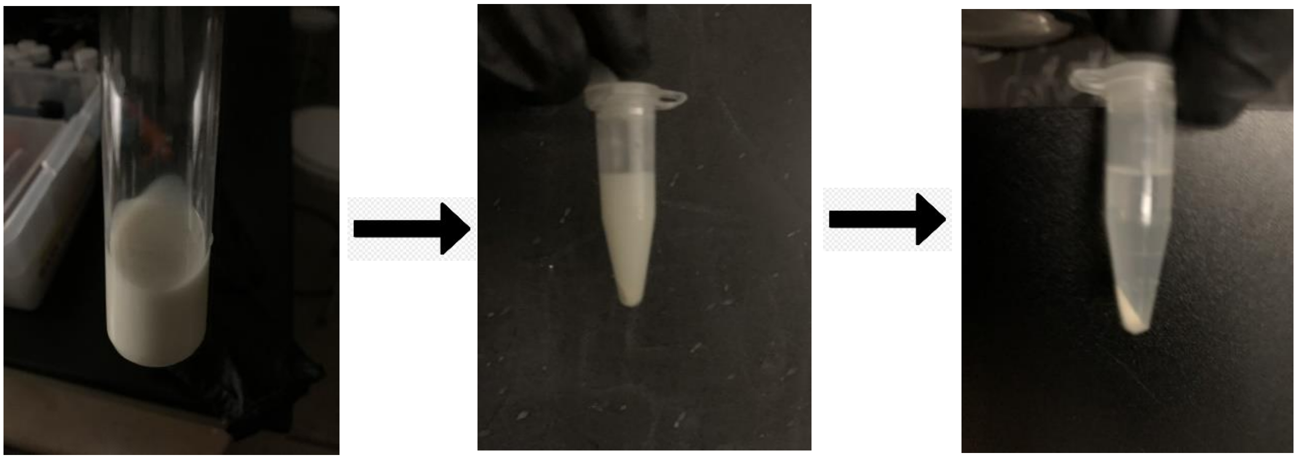

Homogenized mixture of $Sb(Ph)_4(MCO)$ and AgBr in CH_3CN (left panel), then transferred into an Eppendorf tube (middle), and after centrifugation (right panel).

Preparation of Tl(TCO) was carried out using the reaction and procedure shown below:

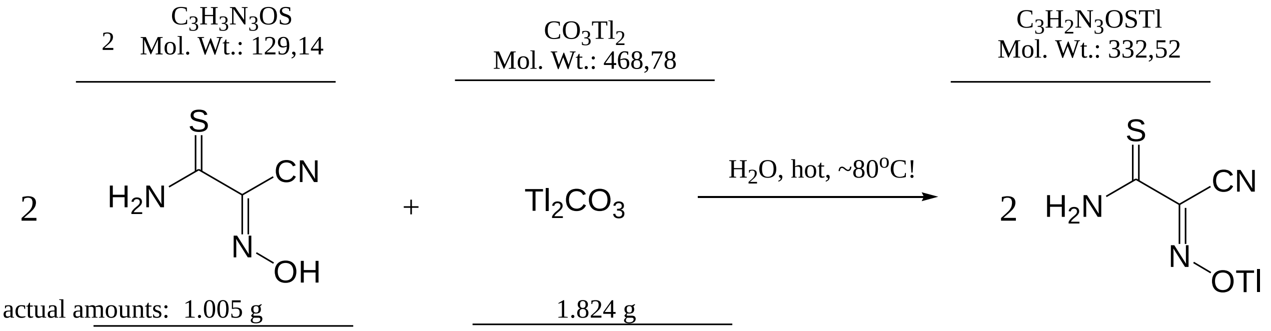

To a solution of 1.005 g (7.78 mM) of H(TCO) in 15 mL of distilled and degassed water at ~80°C in the Schlenk flask under flow of argon a solution of 1.824 g (3.90 mM) of thallium(I) carbonate were added ad once under intense stirring. In 10-15 seconds massive orange precipitate formed. It was filtered, washed with 5 mL of DI water and dried in desiccator giving 3.781 g of Tl(TCO) with 91% yield. Actual appearance of the salt is shown in SI1 above, while results of elemental analysis determination are presented below.

ATLANTIC MICROLAB, INC.

Sample No. Tl(TCO)

6180 Atlantic Blvd. Suite M
Norcross, GA 30071

www.atlanticmicrolab.com

PROFESSOR/SUPERVISOR: Prof. N. Gerasimchuk

PO# / CC#:

SUBMITTER

Company / School Missouri State University

Address 901 South National Avenue

Dept. Chemistry Temple Hall 431

Springfield, MO 65897

NAME Kevin Pinks DATE 3/4/19

PHONE (417) 836-5607 - FAX!

Element	Theory	Found	
C	10.8400	11.07	
H	0.6100	0.44	
N	12.6400	12.35	
S	9.6400	9.74	

Single ☑ Duplicate ☐

Elements Present: C,H,N,O,S, Tl

Analyze for: C,H,N,S

Hygroscopic ☐ Explosive ☐

M.P. ______ B.P. ______

To be dried: Yes ☐ No ☐

Temp. ______ Vac. ______ Time ______

RUSH SERVICE ☐ Rush service guarantees analyses will be completed and results available by 6pm EST on the day the sample is received by 11am.

Include Email Address or Fax # Below

Date Received MAR 07 P.M. Date Completed MAR 08 2019

Remarks:

Actual photographs under microscope of crystalline organoantimony(V) cyanoximates containing thioamide groups: **A** - $SbPh_4$(TCO), **B** - $SbPh_4$(TDCO).

A

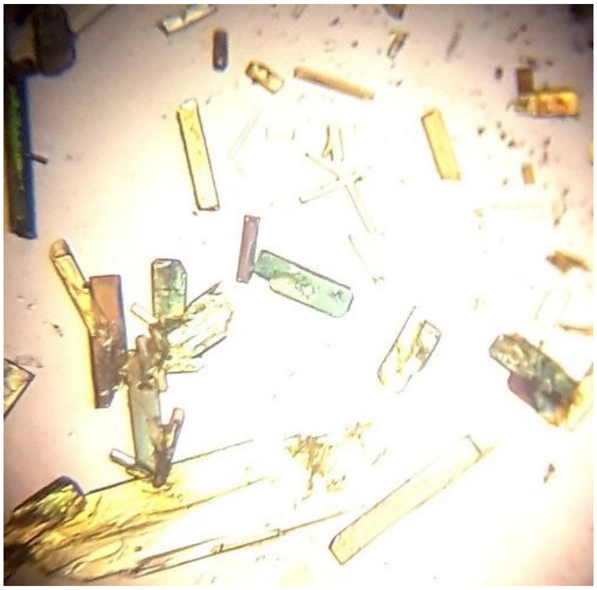

B

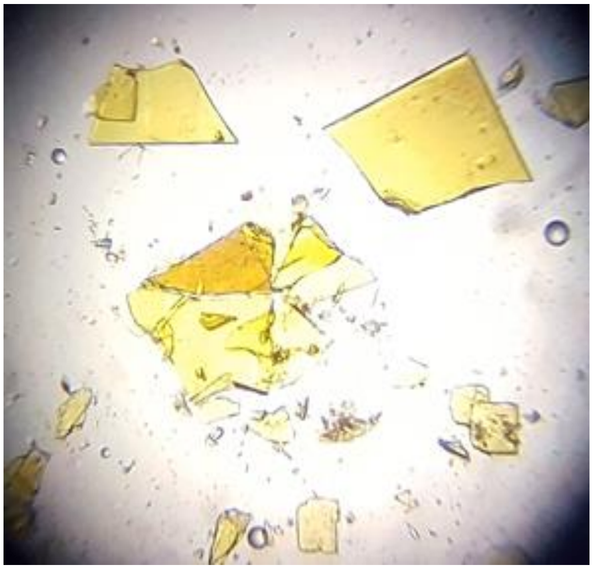

Table S1. Tentative assignment of important vibrational frequencies (cm^{-1}) for synthesized organoantimony(V) cyanoximates.

	vibrations in cyanoxime group							C_6H_5 - group and Sb-Ph vibrations		
Compound	ν (NH_2)	ν (C≡N)	ν (C=X)[a]	ν $(C=N)_{pyr}$	ν $(C=N)_{ox}$	ν (N-O)	ρ (NH_2)	ν_{as}(C-H)	$y(\nu'_{1g})$	$\nu(\nu_8)$
$Sb(Ph)_4$(2PCO)	-	2213	-	1589	1463	1016	-	3094	463, 433	689
$Sb(Ph)_4$(3PCO)	-	2218	-	1574	1473	1065	-	3051	459, 444	687
$Sb(Ph)_4$(4PCO)	-	2216	-	1590	1468	1114	-	3057	454, 442, 431	687
$Sb(Ph)_4$(ACO)	3439, 3366	2222	1679	-	1478	1086	1580	3053	442	688
$Sb(Ph)_4$(ECO)	-	2217	1703	-	1477	1091	-	3052	458, 436	689
$Sb(Ph)_4$(MCO)	-	2223	1629	-	1497	1063	-	3061	443	690
$Sb(Ph)_4$(TCO)	3465, 3341	2220	889	-	1583	1067	1618	3062	450	692
$Sb(Ph)_4$(TDCO)	-	2216	973	-	1524	1069	-	3066	445	688

[a] X = sulfur atom (for TCO^- and $TDCO^-$), oxygen atom (for $2PCO^-$, $3PCO^-$, $4PCO^-$, ACO^-, ECO^-, MCO^-). $_{ox}$ – oxime, $_{pyr}$ – pyridine

The $^{13}C\{^{1}H\}$ NMR spectroscopic results for tetraphenylantimony(V) cyanoximates show expected chemical and magnetic equivalency of all four phenyl rings in structures and values of chemical shifts are tabulated below.

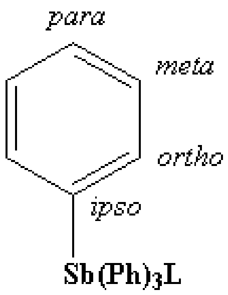

Table S2. Chemical shifts for the $Sb(Ph)_4Br$ (as starting compound) and synthesized organoantimony(V) cyanoximates.

	Assigned (ppm)			
Compound	*ipso*	*ortho*	*meta*	*para*
Sb(Ph)$_4$Br	135.38	135.04	129.44	131.15
$Sb(Ph)_4$(ECO)	133.97	135.57	129.207	136.96
$Sb(Ph)_4$(2PCO)	135.79	135.65	129.02	130.54
$Sb(Ph)_4$(3PCO)	131.53	135.48	129.17	130.75
$Sb(Ph)_4$(4PCO)	129.01	135.54	129.12	149.69
$Sb(Ph)_4$(MCO)	134.9	135.59	129.2	130.9
$Sb(Ph)_4$(TCO)	133.45	135.34	129.52	131.29

Table S3. Chemical shifts values for cyanoxime moieties in the $^{13}C\{^1H\}$ NMR spectra of tetraphenylantimony(V) cyanoximates.

<table>
<tr><th>Compound</th><th>Structure</th><th>δ (ppm)</th></tr>
<tr><td>$Sb(Ph)_4(2PCO)$</td><td>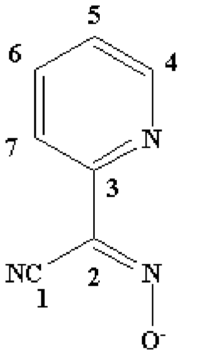
</td><td>C_1 - 112.03
C_2 - 132.38
C_3 - 152.24
C_4 - 148.79
C_5,C_6,C_7- 135.79; 122.46; 118.24</td></tr>
<tr><td>$Sb(Ph)_4(3PCO)$</td><td>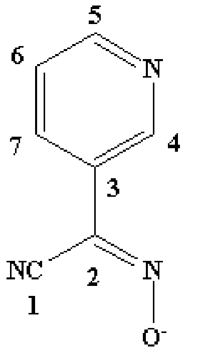
</td><td>C_1 - 111.55
C_2 - 128.52
C_3 - 127.99
C_4 - 149.05
C_5 - 145.76
C_6,C_7 - 129.17; 123.09</td></tr>
<tr><td>$Sb(Ph)_4(4PCO)$</td><td>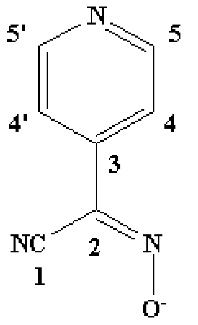
</td><td>C_1 - 111.56
C_2 - 150.15
C_3 - 140.05
$C_{4,4'}$ - 118.24
$C_{5,5'}$ - 149.69</td></tr>
</table>

Table S3 (continued). Chemical shifts values for cyanoxime moieties in the $^{13}C\{^{1}H\}$ NMR spectra of tetraphenylantimony(V) cyanoximates.

Compound	Structure	δ (ppm)
$Sb(Ph)_4$(ECO)	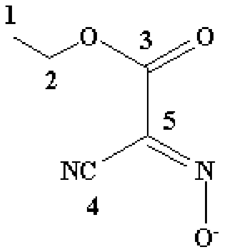	C_1 - 112.03<br>C_2 - 132.38<br>C_3 - 152.24<br>C_4 - 148.79<br>C_5,C_6,C_7 - 135.79; 122.46; 118.27
$Sb(Ph)_4$(MCO)	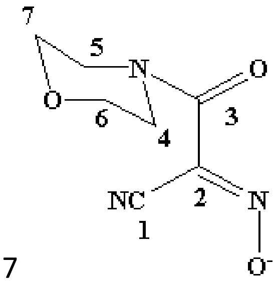 7	C_1 - 127.87<br>C_2 - 111.54<br>C_3 - 159.99<br>C_4 - 46.60<br>C_5 - 43.02<br>C_6 - 66.53<br>C_7 - 68.97
$Sb(Ph)_4$(TCO)<br>***trans-anti***	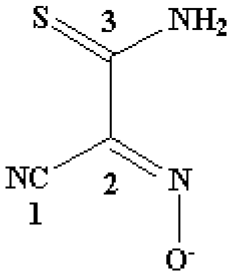	C_1 - 110.92<br>C_2 - 131.63<br>C_3 - 190.01
$Sb(Ph)_4$(TCO)<br>***trans***-*syn*	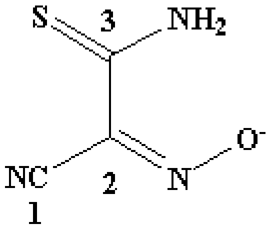	C_1 - 116.92<br>C_2 - 136.38<br>C_3 - 184.98

The $^{13}C\{^1H\}$ NMR spectrum of $SbPh_4(3PCO)$ in CD_2Cl_2 at 295 K after 12,000 repetitions. Shown only *sp*^{2} and *sp* regions of spectrum. Asterisks indicate 4 signals from phenyl groups.

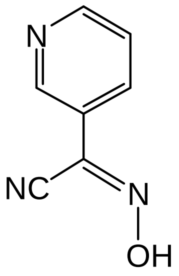

ligand

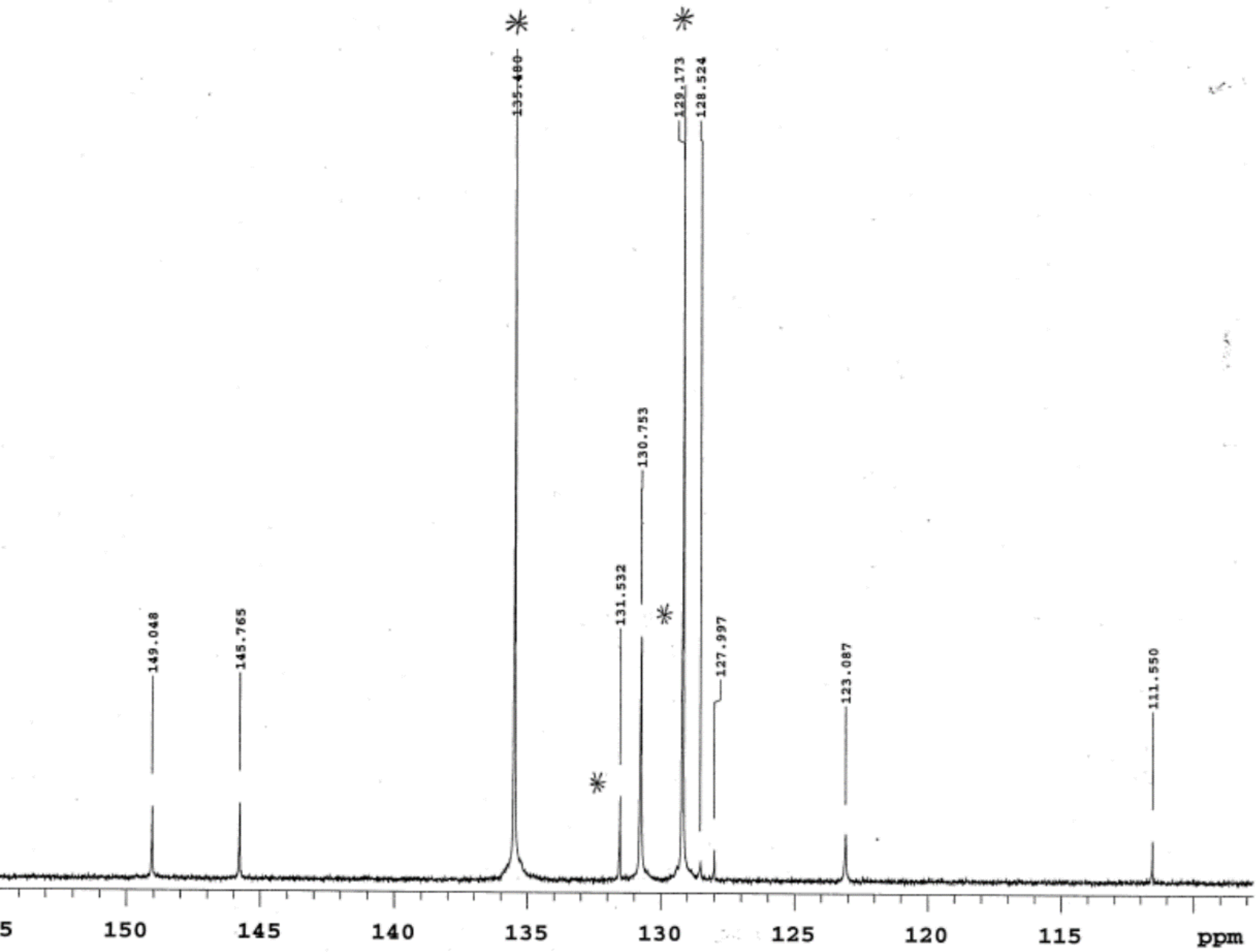

The $^{13}C\{^{1}H\}$ NMR spectrum of $SbPh_4$(2PCO) in CD_2Cl_2 at 298 K after 20,000 repetitions. Shown full spectrum including solvent peaks. Asterisks indicate 4 signals from phenyl groups.

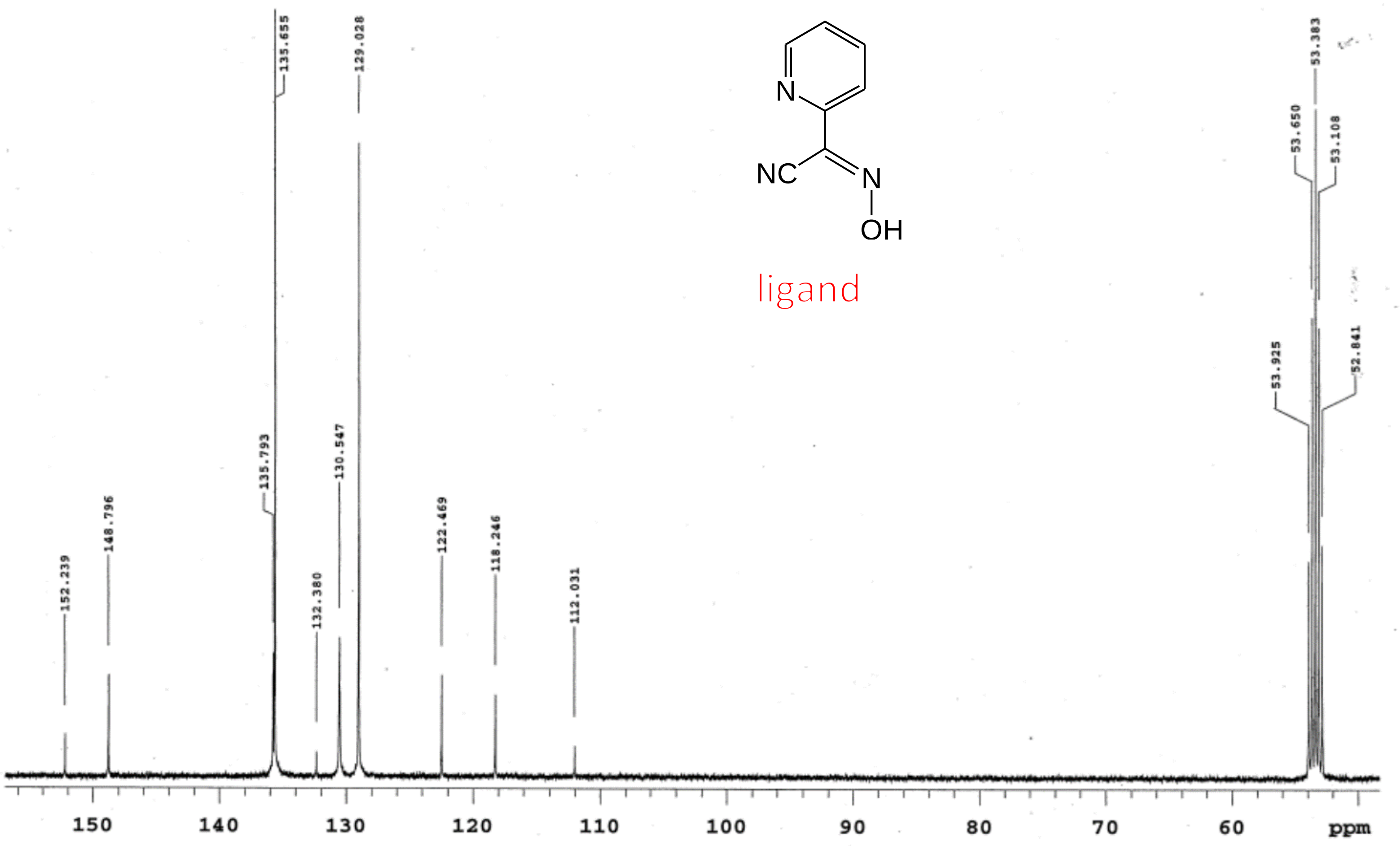

The $^{13}C\{^1H\}$ NMR spectrum of $SbPh_4(ECO)$ in CD_2Cl_2 at 298 K after 14,000 repetitions. Shown full spectrum including solvent peaks. Asterisks indicate 4 signals from phenyl groups.

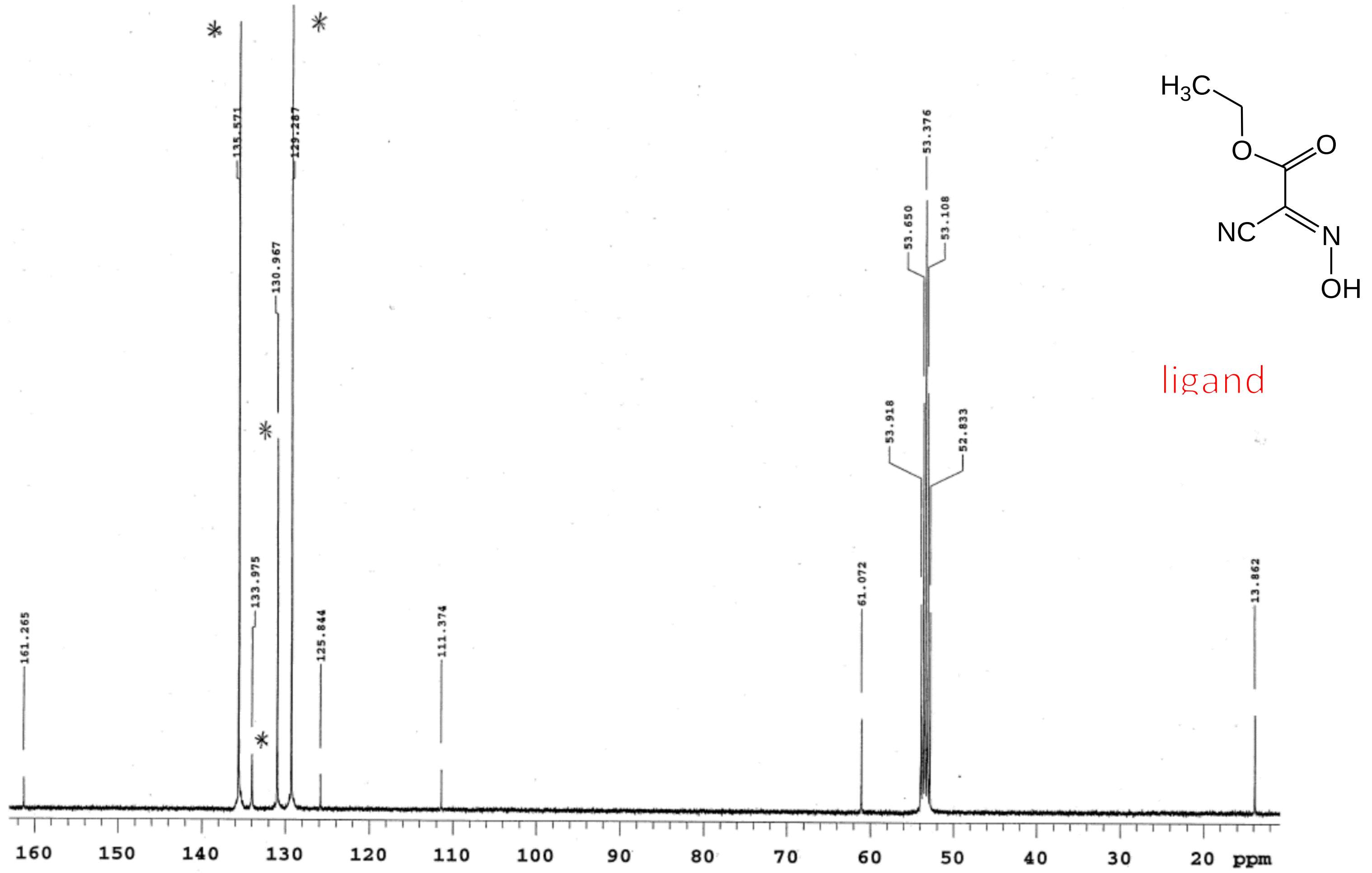

Actual videomicroscope images of single crystals of organoantimony(V) compounds used for structures determination.

Face-indexed crystal od **$SbPh_4$(2PCO)**.

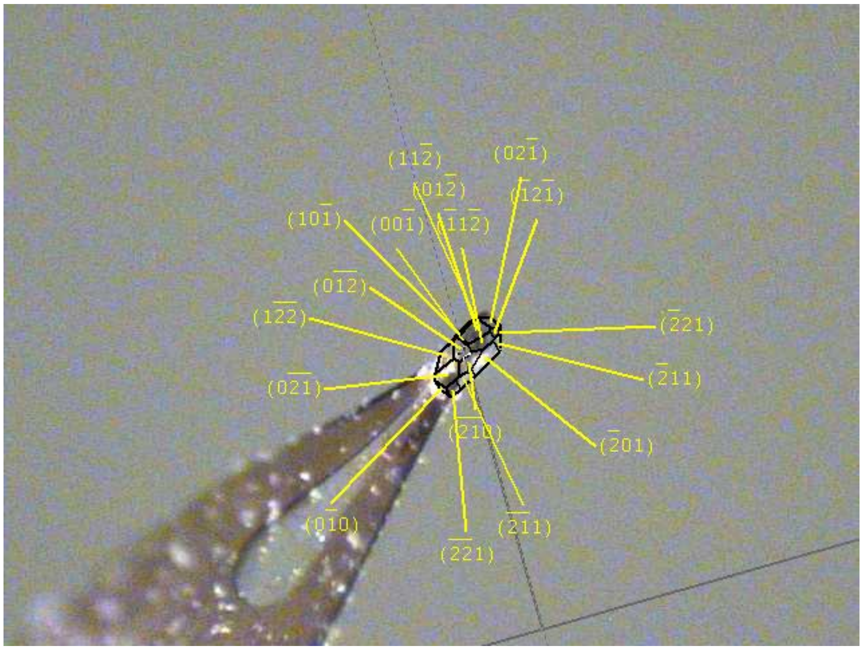

Face-indexed crystal of **$SbPh_4$(4PCO)**.

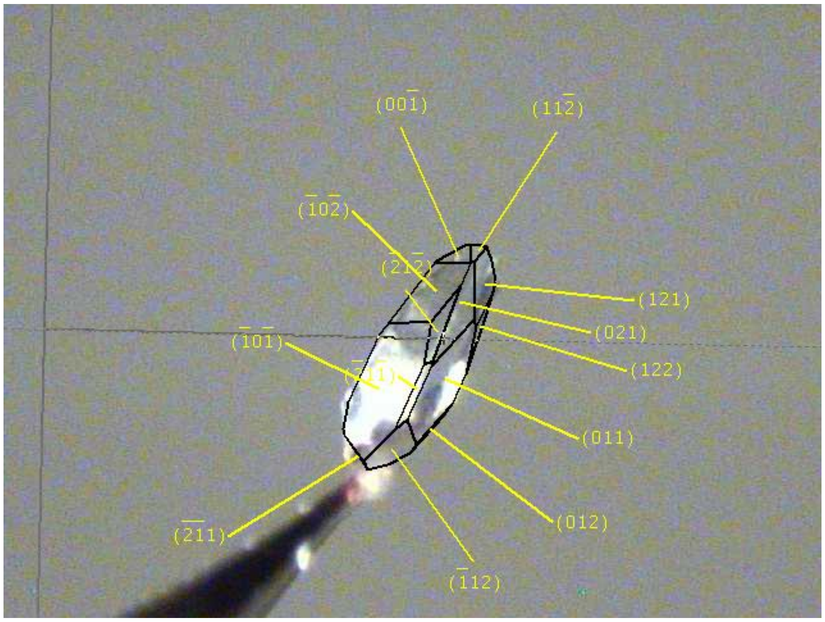

Actual videomicroscope images of single crystals of organoantimony(V) compounds used for structures determination.

Face-indexed crystal od **$SbPh_4$(3PCO)**.

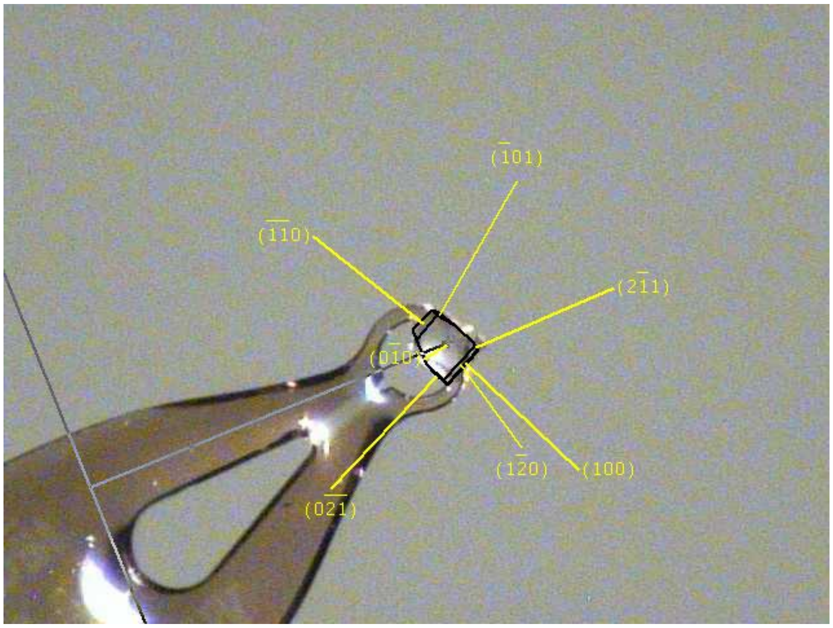

Face-indexed crystal od **$SbPh_4$(TCO)**.

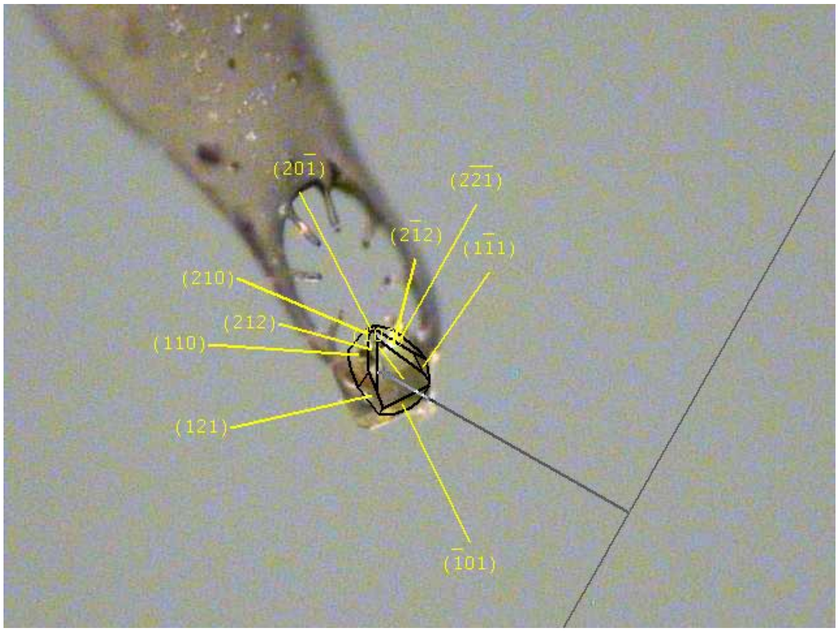

Geometry of coordination polyhedron of Sb(V) in the structure of **$SbPh_4$(3PCO)**.

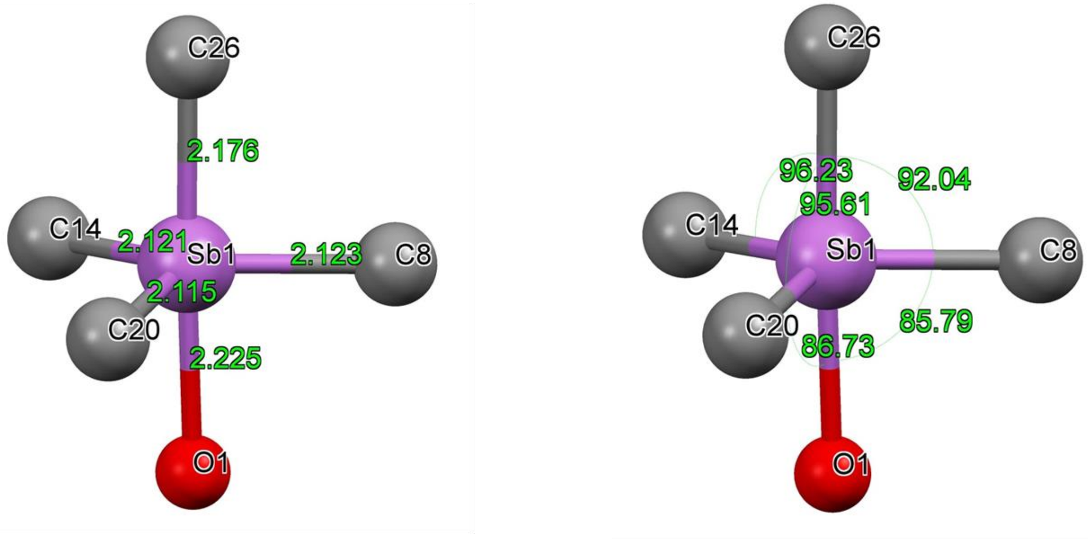

bonds angles

angle C26-Sb1-O1 = 177.38°

Weak C-H---N interactions stabilizing planar geometry of the cyanoxime in the compound with two atoms shown in space-fill representation.

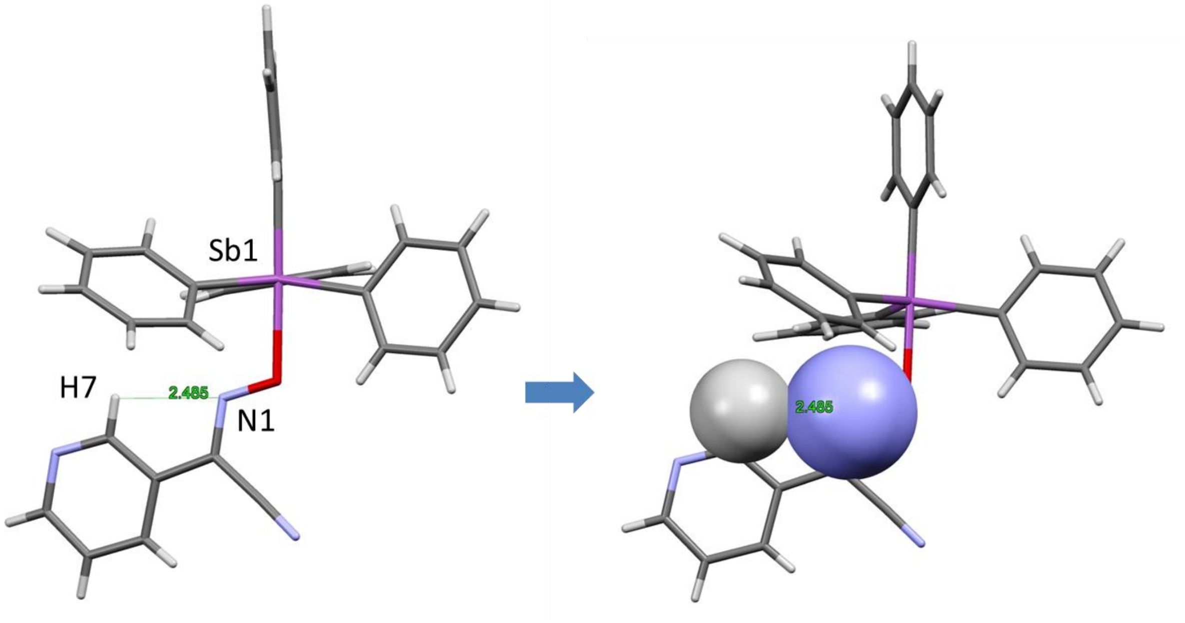

Geometry of coordination polyhedron of Sb(V) in the structure of **$SbPh_4$(ACO)**.

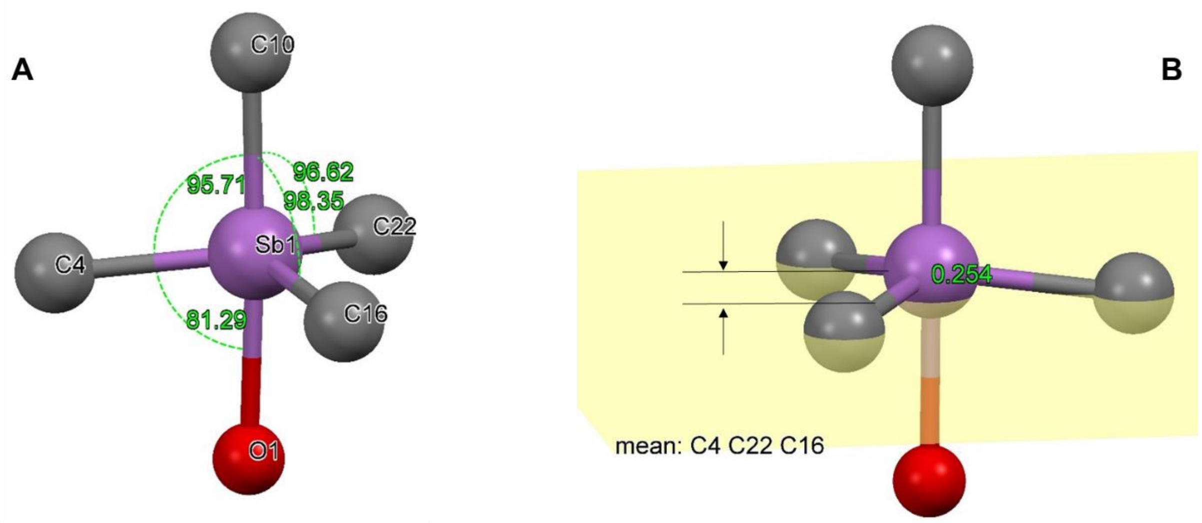

Weak N-H---N interaction stabilizing planar geometry of the cyanoxime in the compound with two atoms shown in space-fill representation emphasizing electrostatic contact.

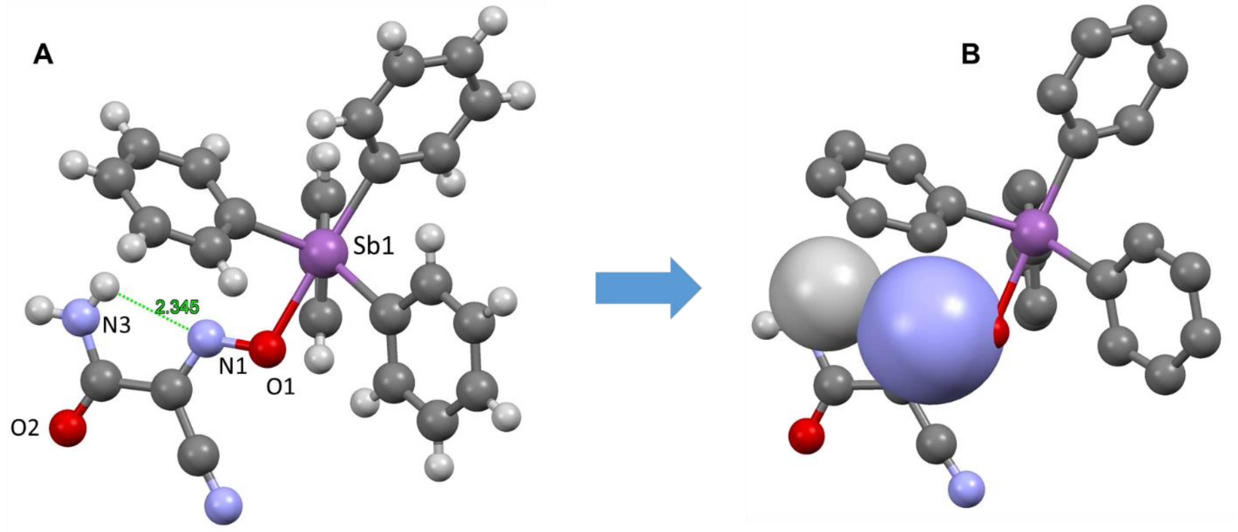

Top panel: molecular structure and principal atoms numbering scheme in the structure of **$SbPh_4$(ECO)** showing two crystallographically different molecules **A** and **B**. The H-atoms are omitted for clarity.

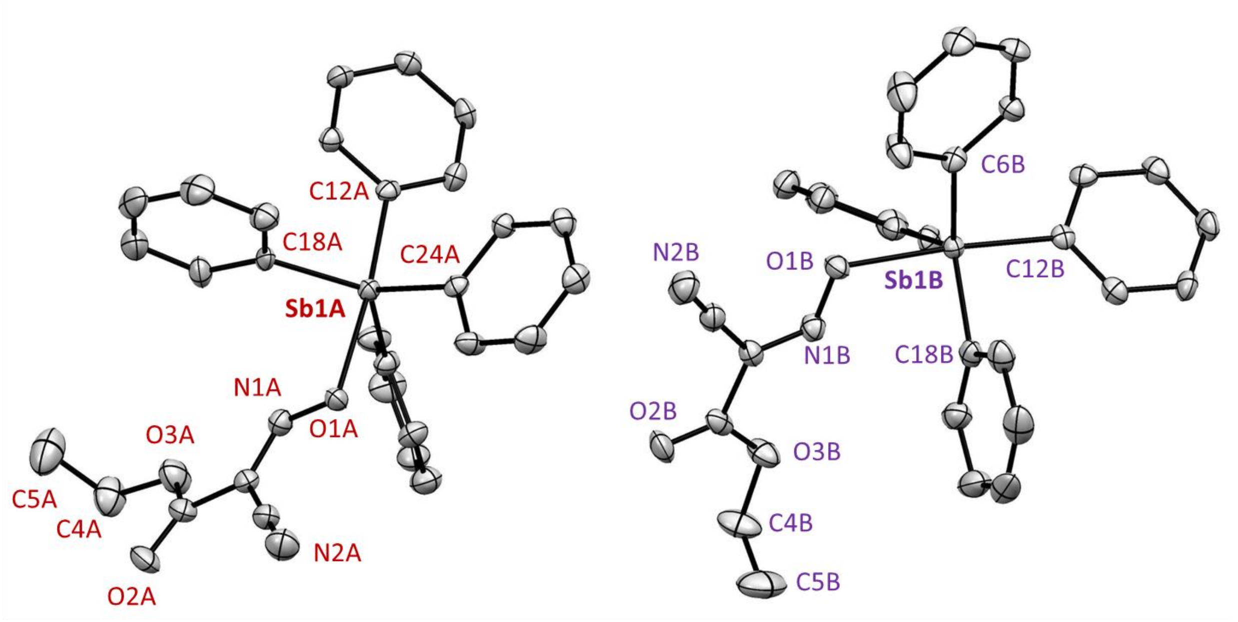

Bottom panel: details of packing of two crystallographically different molecules A and B into the unit cell.

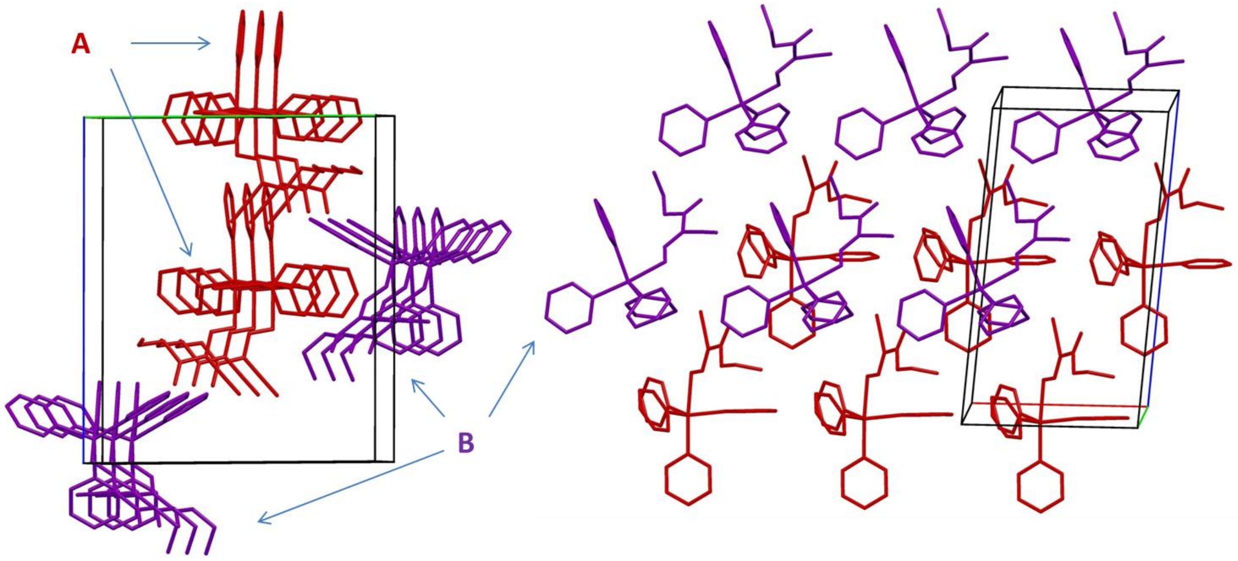

Geometry of coordination polyhedrons of Sb(V) in the structure of **$SbPh_4$(ECO)**: top panel for crystallographically independent molecule **A**, bottom panel for independent molecule **B**.

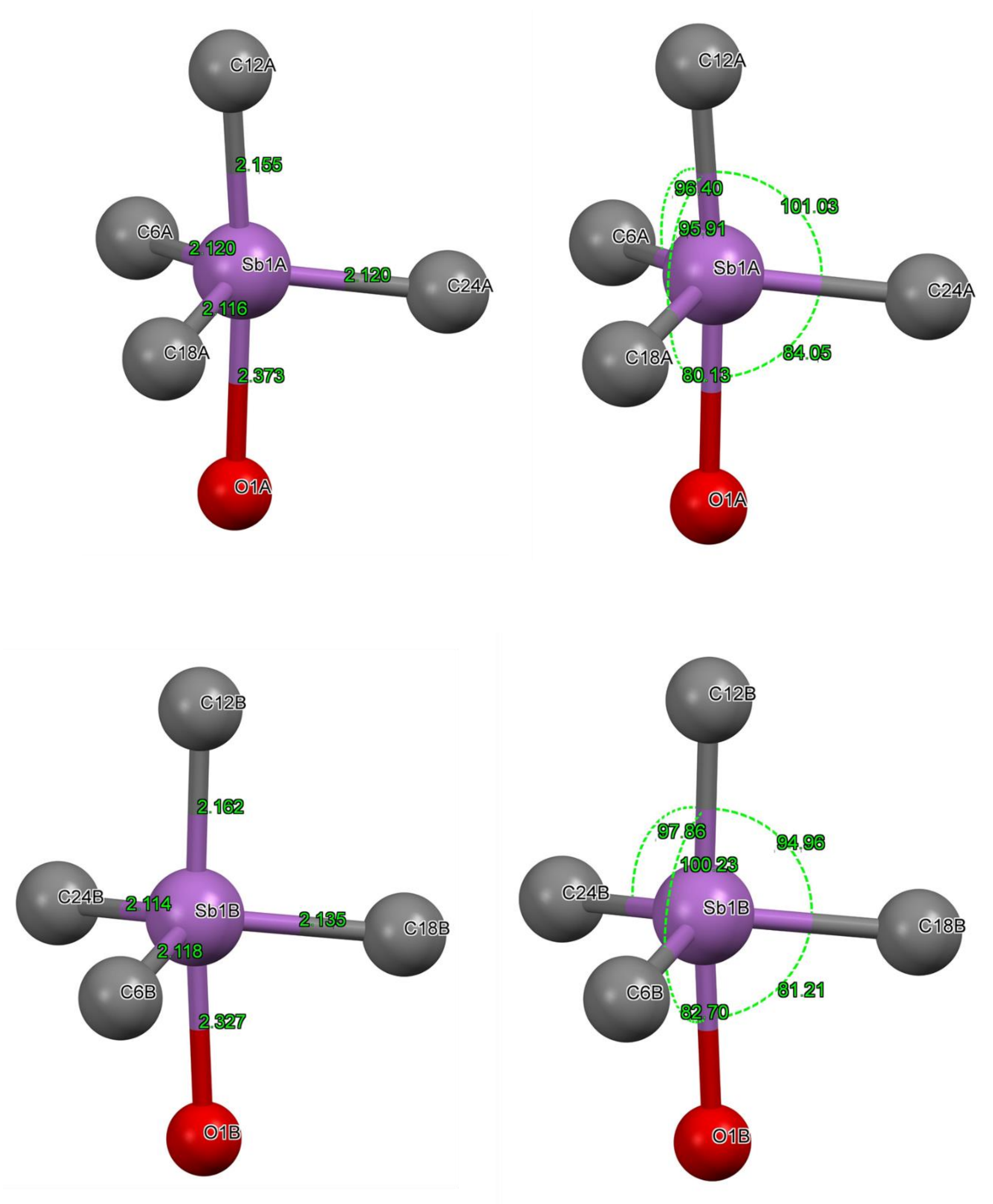

Molecular structure and principal atoms numbering scheme in the structure of **$SbPh_4$(MCO)** showing two crystallographically different molecules **A** and **B**. The H-atoms are omitted for clarity.

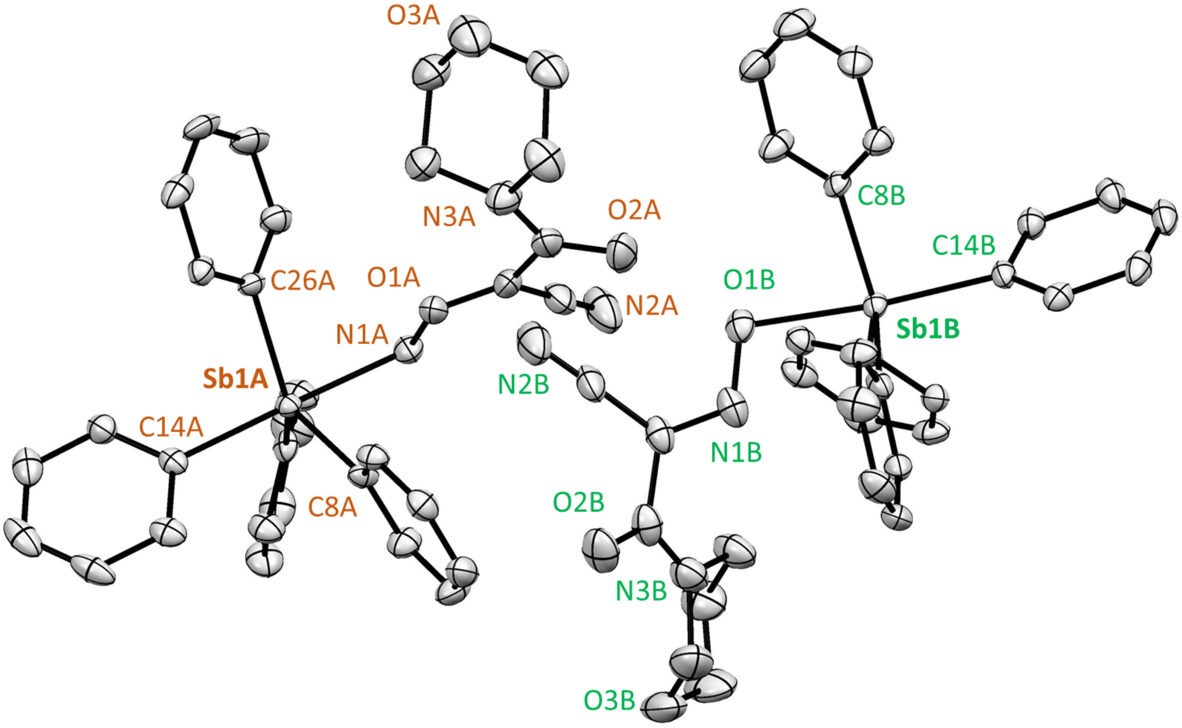

Geometry of coordination polyhedrons of Sb(V) in the structure of **$SbPh_4$(MCO)**: top panel for crystallographically independent molecule **A**, bottom panel for independent molecule **B**.

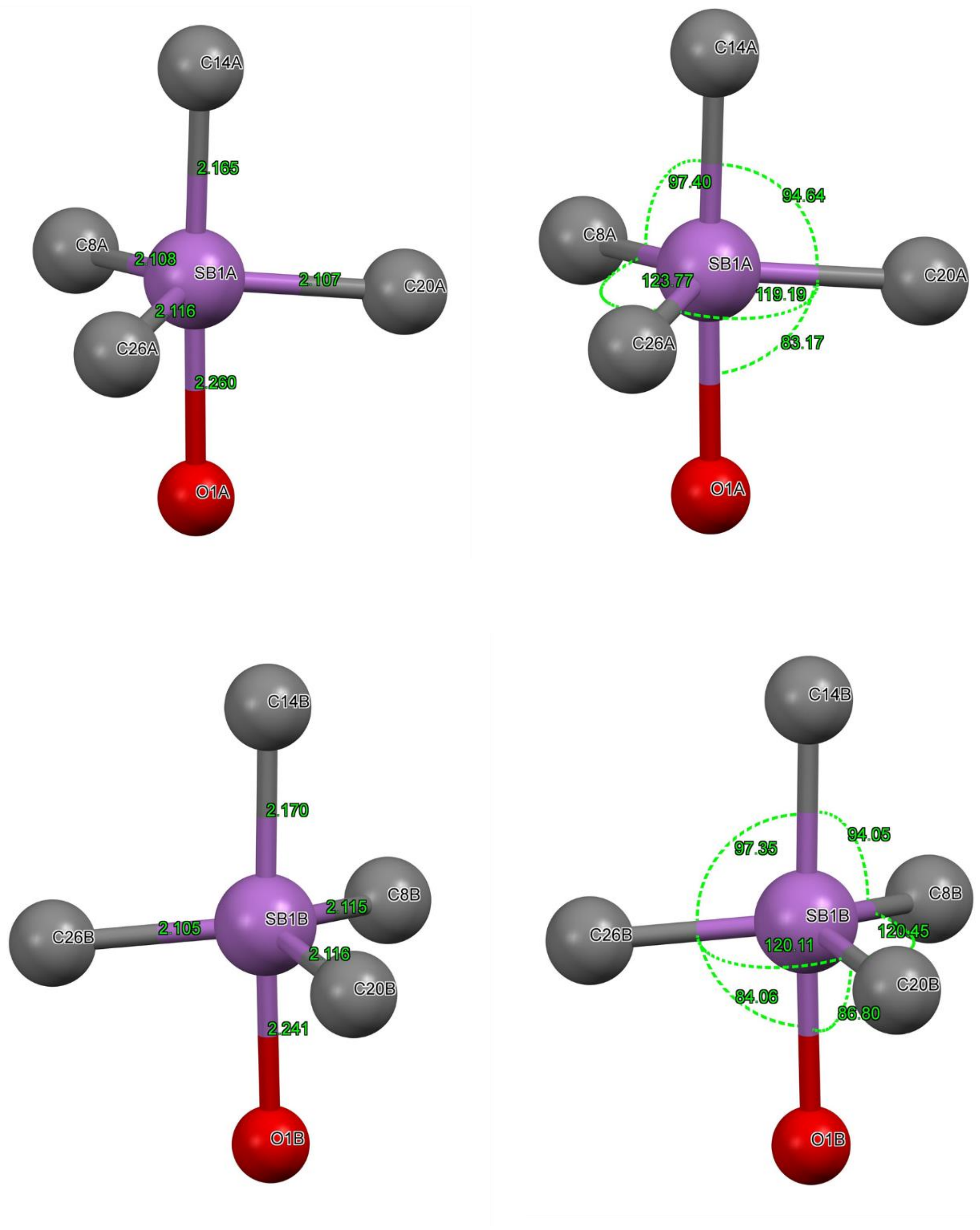

The ASU in the structure of **$SbPh_4$(ECO)** with two crystallographically independent molecules **A** and **B** shown in ORTEP representation at 50% thermal ellipsoids probability level.

Note that the main text presents structure of only one molecule!

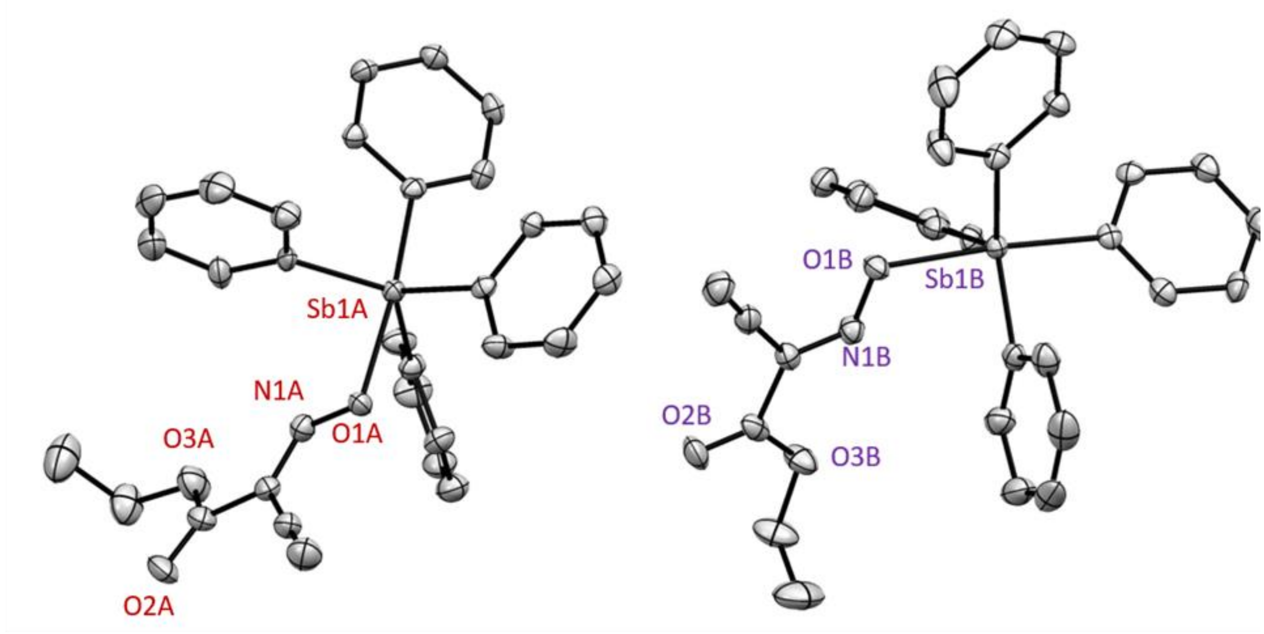

The ASU in the structure of **SbPh$_4$(MCO)** contains two crystallographically independent molecules **A** and **B**.

Note that the main text presents structure of only one molecule!

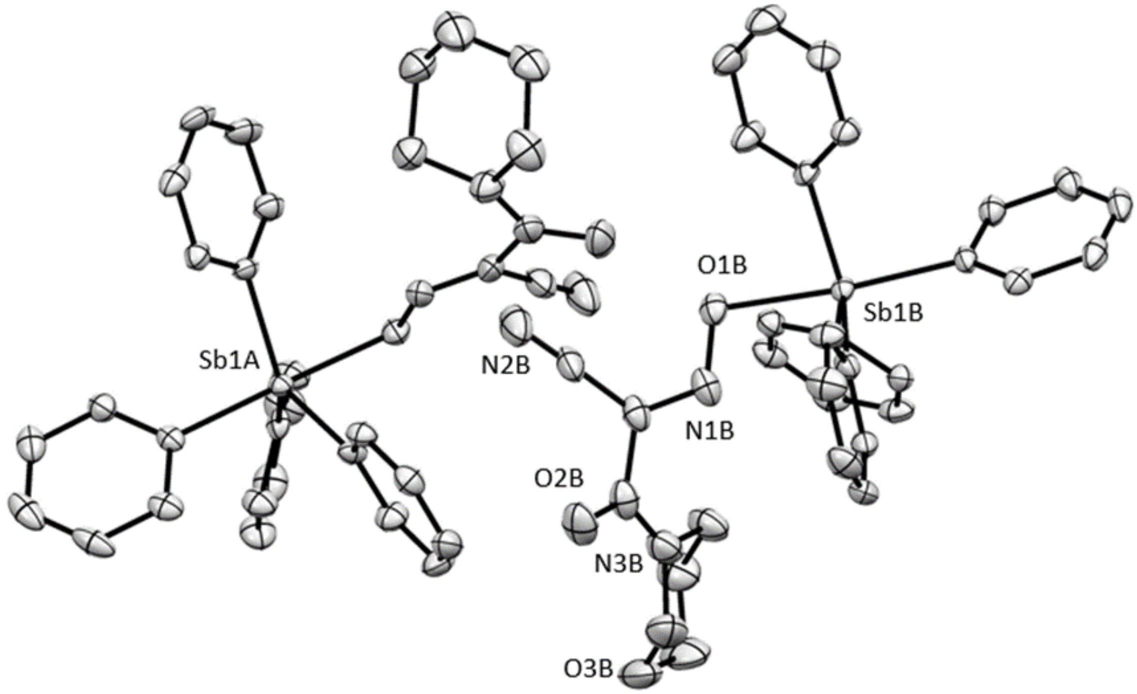

Molecular structure and principal atoms numbering scheme for **$SbPh_4$(TCO)**. This is polymorph I which comprised of co-crystallized mixture of two diastereomers: *syn*- and *anti*-.

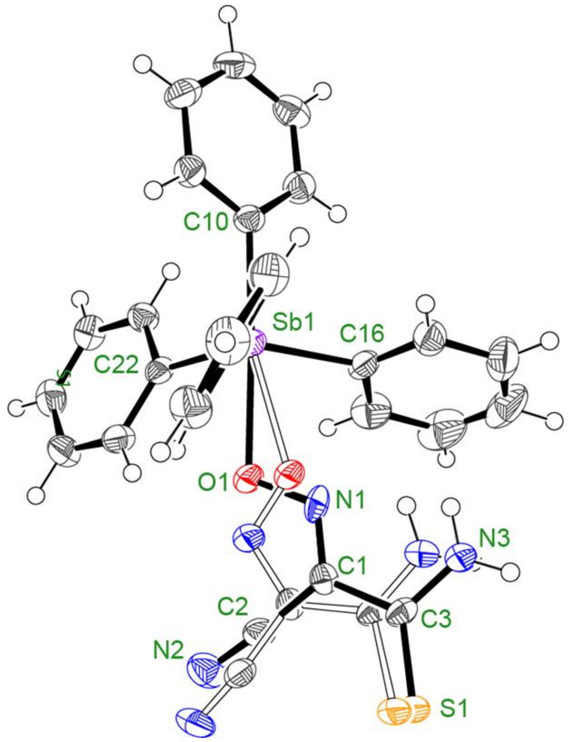

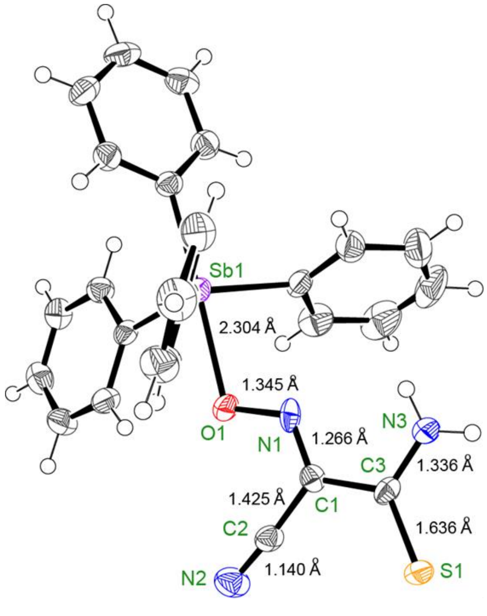

+

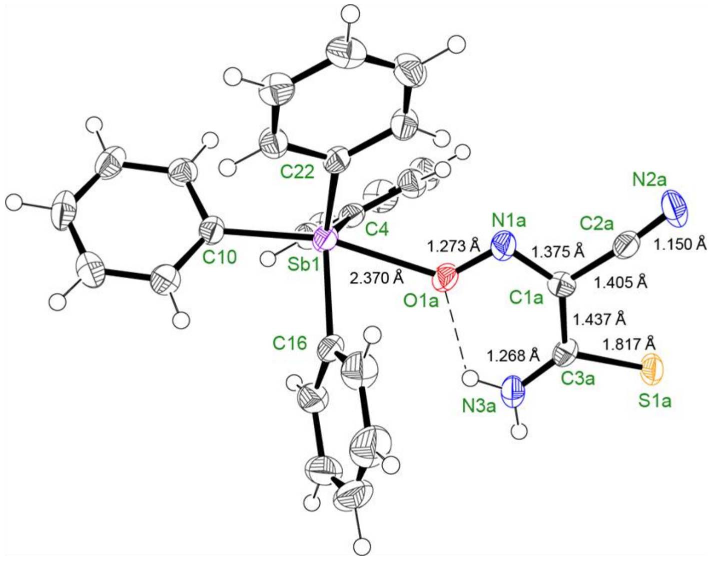

trans-anti isomer, 81.7%

trans-syn isomer, 18.3%

Typical traces of weight loss (green) and heat flow (blue) for **$SbPh_4$(ECO)** selected as example showing the melting point of the compound at 144°C followed by its decomposition. Table below summarizes results of TG/DSC measurements for all synthesized tetraphenylantimony(V) cyanoximates.

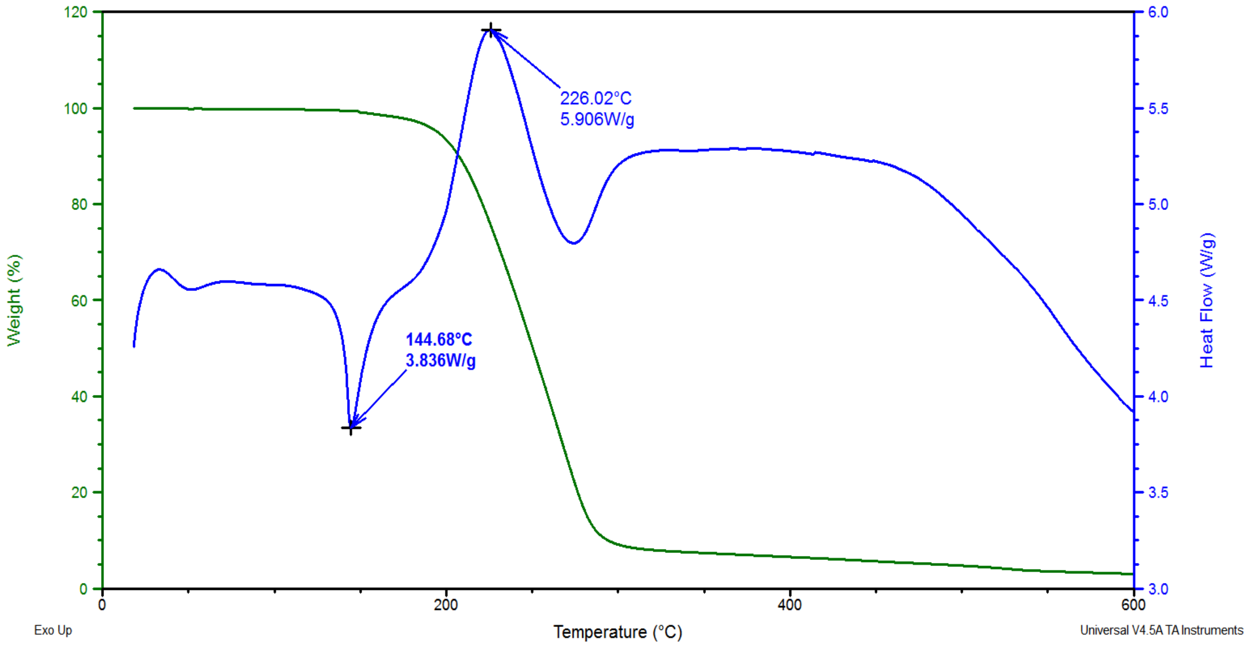

Compound		Events, temperatures (°C)
$SbPh_4$(2PCO)·H_2O*	weight loss for ~1 H_2O at ~166	*endo-*, melting at 204.2; decomposition, *exo-* at 249
$SbPh_4$(3PCO)		*endo-*, melting at 146.9; decomposition, *exo-* at 199
$SbPh_4$(4PCO)		*endo-*, melting at 145.4; decomposition, *exo-* at 224 and second decomposition, *exo-* at 283
$SbPh_4$(ACO)		*endo-*, melting at 199.5; decomposition, *exo-* at 239
$SbPh_4$(ECO)		*endo-*, melting at 144.7; decomposition, *exo-* at 226
$SbPh_4$(MCO)		*endo-*, melting at 175.2; decomposition, *exo-* at 238
$SbPh_4$(TCO)		*endo-*, melting at 185.6; decomposition, *endo-* at 239; and second decomposition, *endo-* at 286
$SbPh_4$(TDCO)		*endo-*, melting at 148.7; decomposition, *exo-* at 221

*- X-ray structure gives evidence of anhydrous behavior. Therefore, the complex most likely absorbed moisture from handling.

Observed correlations between acidity of the free cyanoxime HL and chemical shift of the *ipso* carbon in NMR spectra of organoantimony(V) cyanoximates.

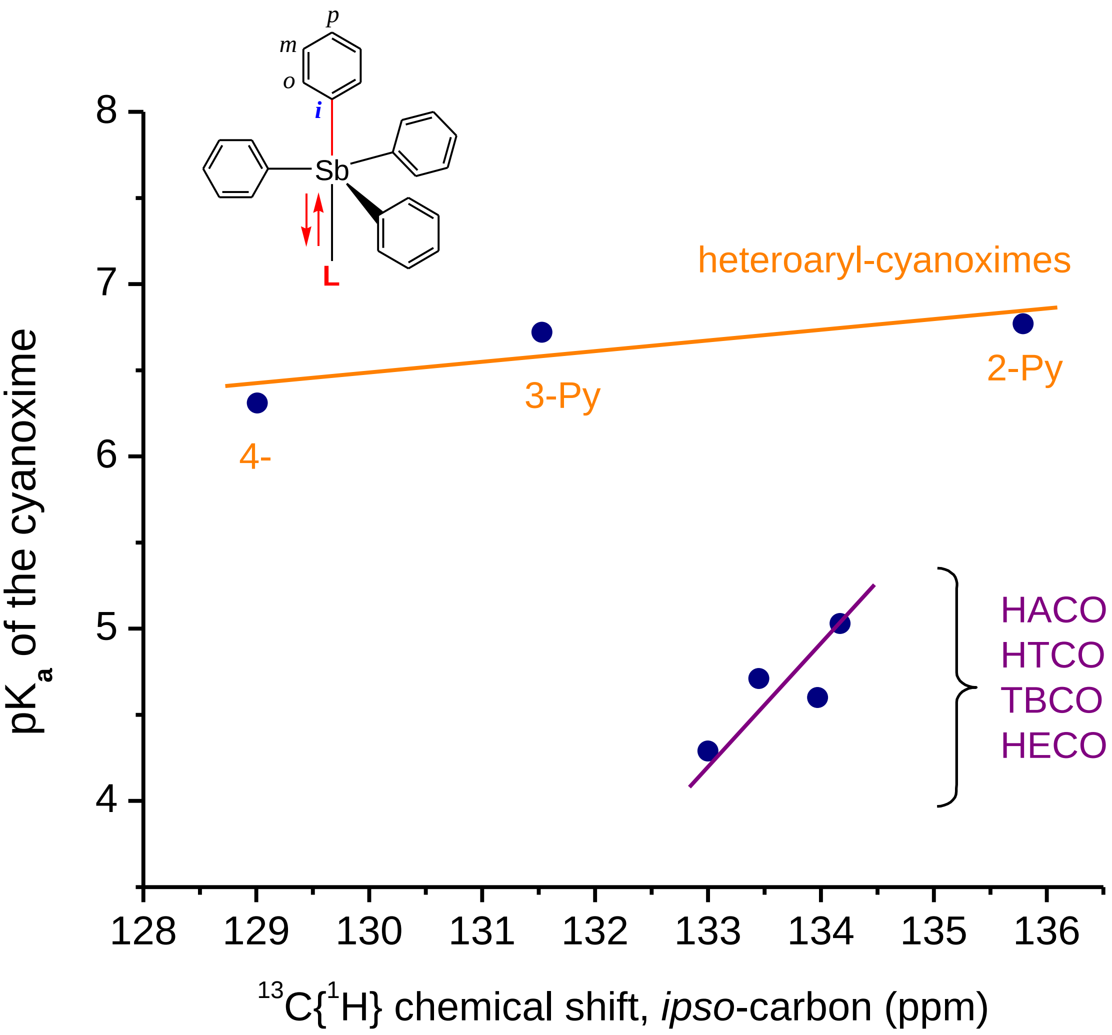

The clearance zones determined by the disc diffusion assay. The black bars represent undiluted, dark grey and light grey - x2 and x4 diluted antibacterial compounds. Controls and test compounds that showed no clearance are not included; p-value: *≤ 0.1, **≤ 0.05

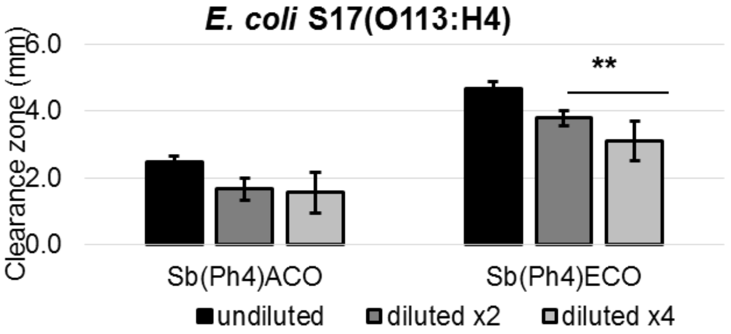

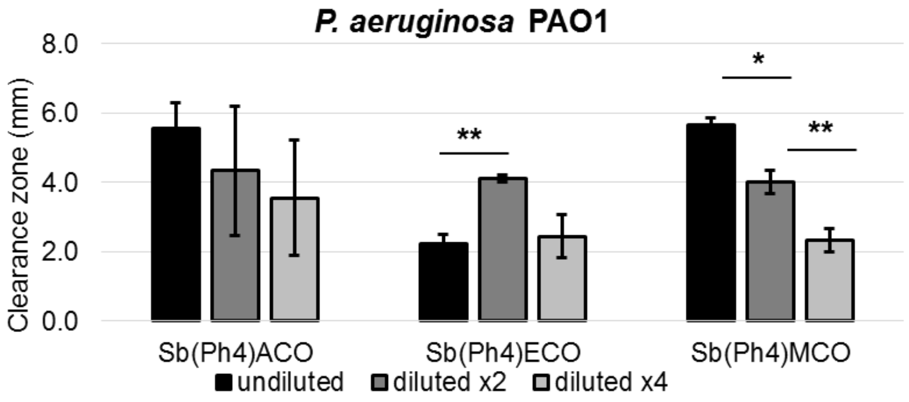

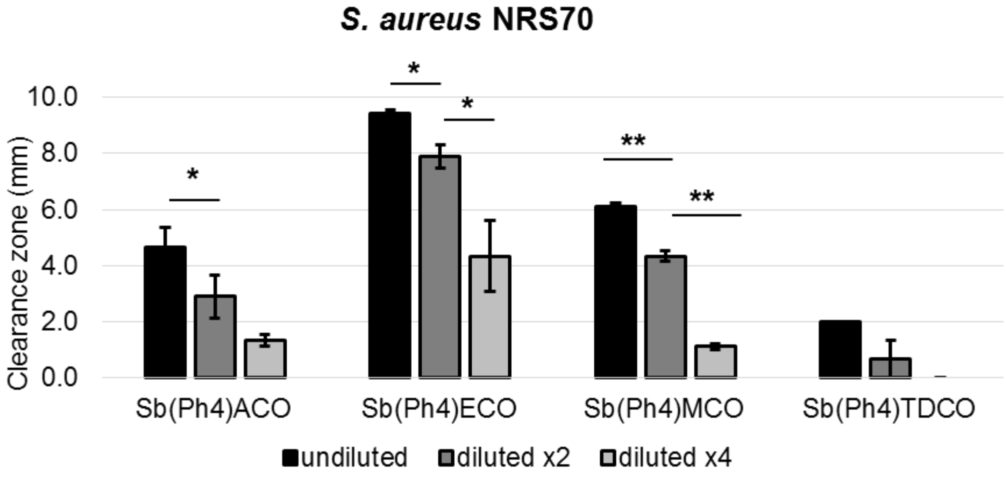

The clearance zones determined by the disc diffusion assay. The black bars represent undiluted, dark grey and light grey - x2 and x4 diluted antibacterial compounds. Controls are not included. Data shown are means + SEM for 3 individual experiments.

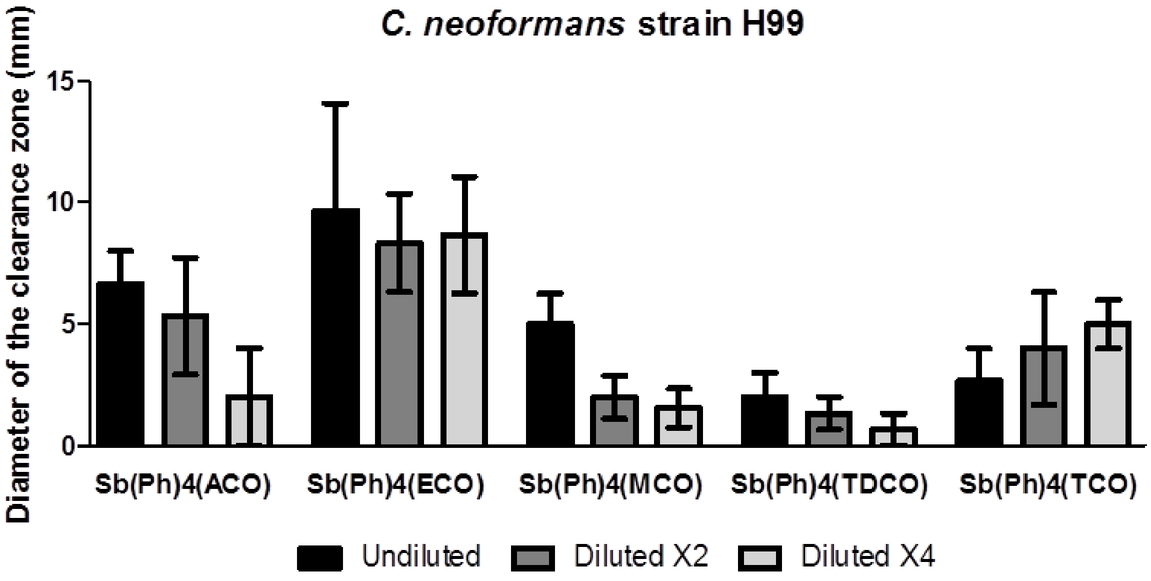

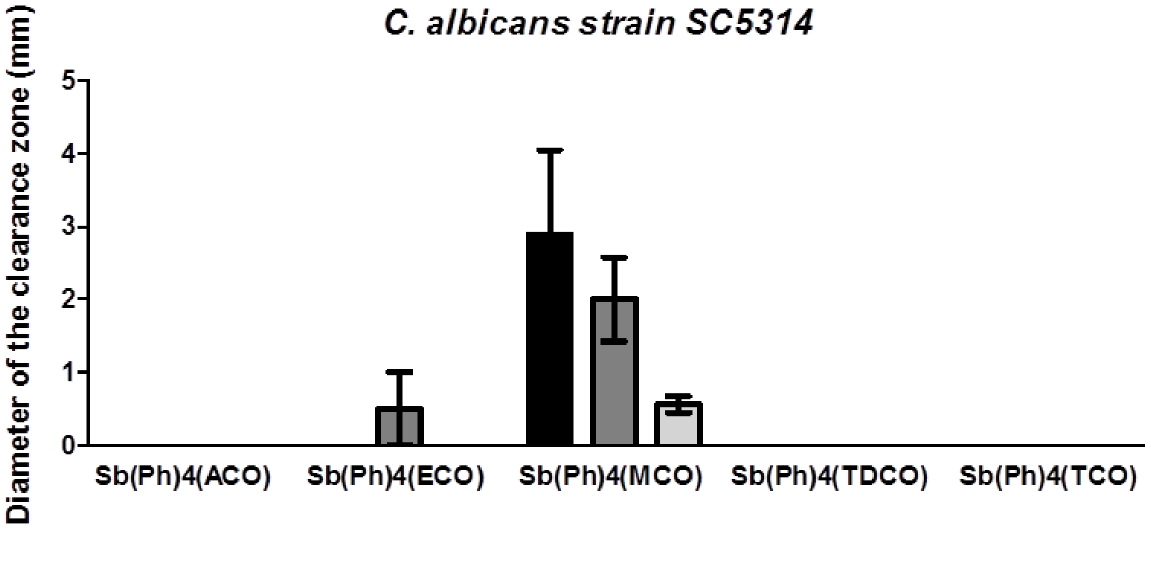

Representative images of discs with undiluted compounds subjected to the disc diffusion assay. Green and orange represent test compounds and ligand controls, respectively. Clearance zones are pointed at with the red arrows.

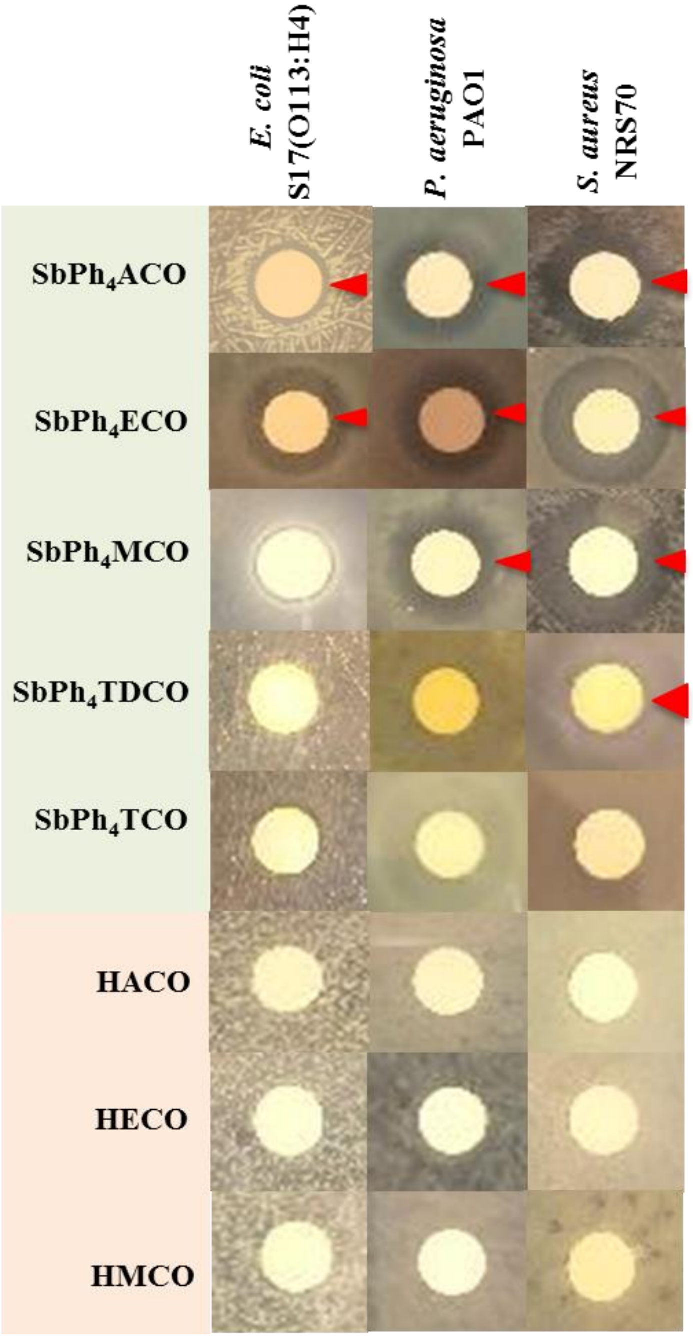

Representative images of discs with undiluted compounds subjected to the disc diffusion assay. Green and orange represent test compounds and ligand controls, respectively. Clearance zones are pointed at with the red arrows.

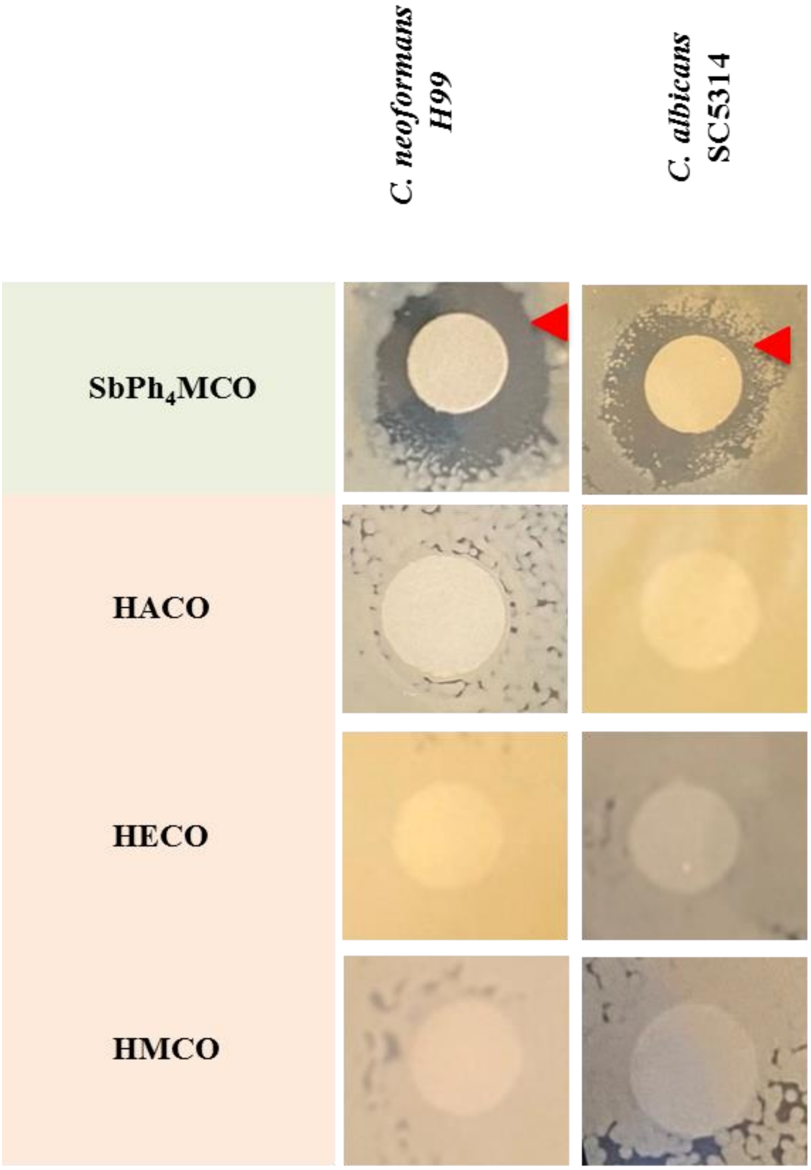

checkCIF/PLATON REPORTS (basic structural check)

Structure factors have been supplied for datablock(s) Sb-Ph4-2pco

No syntax errors found.
Please wait while processing ....

CIF dictionary
Interpreting this report

Structure factor report

Datablock: Sb-Ph4-2PCO

Bond precision: C-C = 0.0044 A Wavelength=0.71073

Cell:	a=12.6027(8)	b=14.4415(10)	c=14.6694(10)
	alpha=90	beta=104.9644(10)	gamma=90

Temperature: 100 K

	Calculated	Reported
Volume	2579.3(3)	2579.3(3)
Space group	P 21/n	P 21/n
Hall group	-P 2yn	-P 2yn
Moiety formula	C31 H24 N3 O Sb	C31 H24 N3 O Sb
Sum formula	C31 H24 N3 O Sb	C31 H24 N3 O Sb
Mr	576.29	576.28
Dx,g cm-3	1.484	1.484
Z	4	4
Mu (mm-1)	1.098	1.098
F000	1160.0	1160.0
F000'	1158.02	
h,k,lmax	15,18,18	15,18,18
Nref	5461	5458

Tmin,Tmax 0.846,0.912 0.872,0.949

Tmin' 0.829

Correction method= # Reported T Limits:
Tmin=0.872 Tmax=0.949 AbsCorr = NUMERICAL

Data completeness= 0.999 Theta(max)= 26.710

R(reflections)= 0.0329(4414) wR2(reflections)= 0.0792(5458)

S = 1.039 Npar= 421

The following ALERTS were generated. Each ALERT has the format

test-name_ALERT_alert-type_alert-level.

Click on the hyperlinks for more details of the test.

Alert level C

PLAT094_ALERT_2_C Ratio of Maximum / Minimum Residual Density .... 3.47 Report

PLAT245_ALERT_2_C U(iso) H7 Smaller than U(eq) C7 by 0.013 Ang**2

PLAT245_ALERT_2_C U(iso) H24 Smaller than U(eq) C24 by 0.012 Ang**2

PLAT350_ALERT_3_C Short C-H (X0.96,N1.08A) C22 - H22 . 0.84 Ang.

PLAT975_ALERT_2_C Check Calcd Resid. Dens. 1.06A From O1 0.43 eA-3

Alert level G

PLAT164_ALERT_4_G Nr. of Refined C-H H-Atoms in Heavy-Atom Struct. 24 Note

PLAT883_ALERT_1_G No Info/Value for _atom_sites_solution_primary . Please Do !

PLAT912_ALERT_4_G Missing # of FCF Reflections Above STh/L= 0.600 3 Note

PLAT965_ALERT_2_G The SHELXL WEIGHT Optimisation has not Converged Please Check

PLAT978_ALERT_2_G Number C-C Bonds with Positive Residual Density. 7 Info

---

PLATON version of 04/06/2020; check.def file version of 02/06/2020

Databloc Sb-Ph4-2PCO - ellipsoid plot

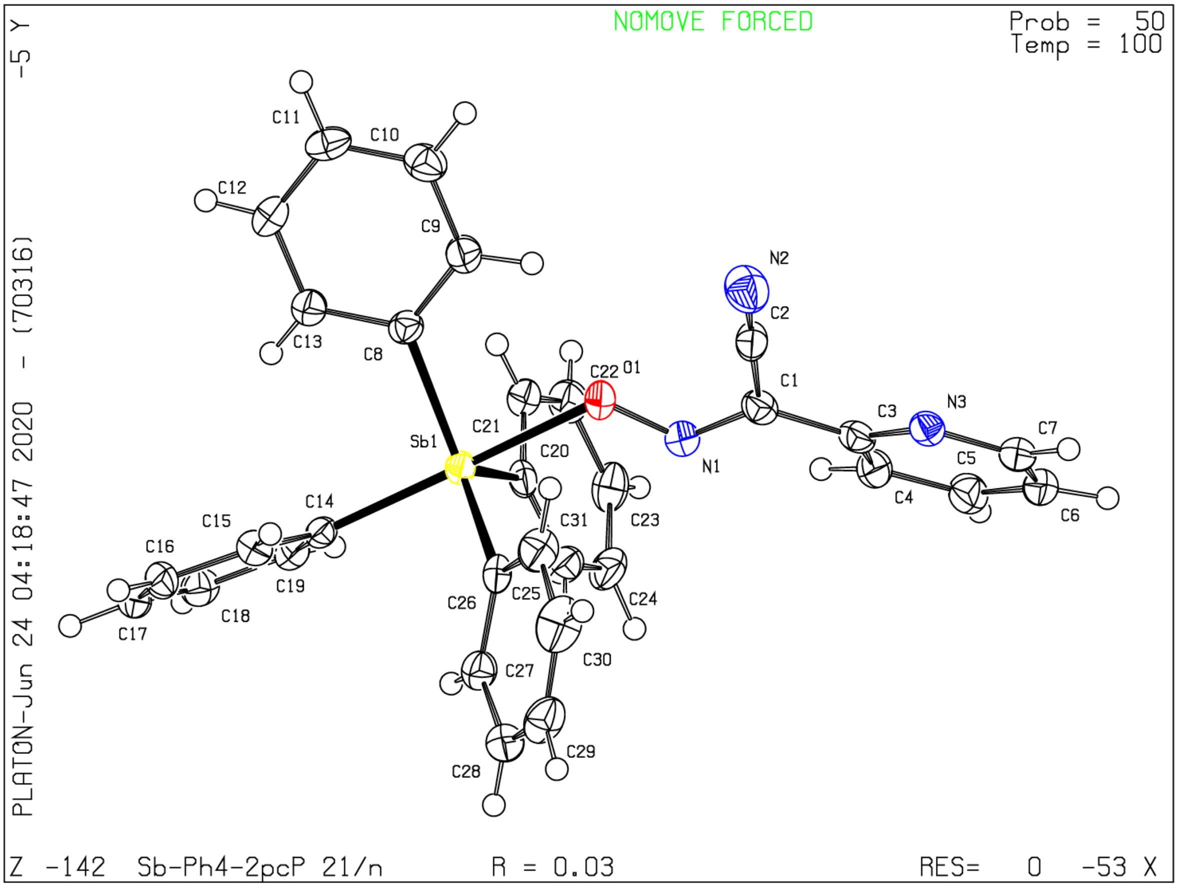

checkCIF/PLATON (basic structural check)

Structure factors have been supplied for datablock(s) Sb-Ph4-3PCO

No syntax errors found.
Please wait while processing ....

CIF dictionary
Interpreting this report

Structure factor report

Datablock: Sb-Ph4-3PCO

Bond precision: C-C = 0.0036 A   Wavelength=0.71073

Cell: a=10.0228(4) b=10.4598(4) c=13.8258(5)
alpha=69.324(1) beta=72.086(1) gamma=83.971(1)

Temperature: 296 K

	Calculated	Reported
Volume	1290.34(9)	1290.34(9)
Space group	P -1	P -1
Hall group	-P 1	-P 1
Moiety formula	C31 H24 N3 O Sb	C31 H24 N3 O Sb
Sum formula	C31 H24 N3 O Sb	C31 H24 N3 O Sb
Mr	576.29	576.28
Dx,g cm-3	1.483	1.483
Z	2	2
Mu (mm-1)	1.098	1.098
F000	580.0	580.0
F000'	579.01	
h,k,lmax	13,13,18	13,13,18
Nref	6224	6218
Tmin,Tmax	0.822,0.897	0.697,0.747
Tmin'	0.798	

Correction method= # Reported T Limits:

Tmin=0.697 Tmax=0.747 AbsCorr = MULTI-SCAN

Data completeness= 0.999 Theta(max)= 27.999

R(reflections)= 0.0242(5636)   wR2(reflections)= 0.0585(6218)

S = 1.055   Npar= 421

The following ALERTS were generated. Each ALERT has the format

test-name_ALERT_alert-type_alert-level.

Click on the hyperlinks for more details of the test.

Alert level C

PLAT230_ALERT_2_C Hirshfeld Test Diff for C11 --C12 . 6.5 s.u.

PLAT241_ALERT_2_C High 'MainMol' Ueq as Compared to Neighbors of C5 Check

PLAT242_ALERT_2_C Low 'MainMol' Ueq as Compared to Neighbors of C3 Check

PLAT350_ALERT_3_C Short C-H (X0.96,N1.08A) C4 - H4 . 0.83 Ang.

And 2 other PLAT350 Alerts

PLAT350_ALERT_3_C Short C-H (X0.96,N1.08A) C12 - H12 . 0.84 Ang.

PLAT350_ALERT_3_C Short C-H (X0.96,N1.08A) C19 - H19 . 0.84 Ang.

PLAT911_ALERT_3_C Missing FCF Refl Between Thmin & STh/L= 0.600 2 Report

---

Alert level G

PLAT154_ALERT_1_G The s.u.'s on the Cell Angles are Equal ..(Note) 0.001 Degree

PLAT164_ALERT_4_G Nr. of Refined C-H H-Atoms in Heavy-Atom Struct. 24 Note

PLAT230_ALERT_2_G Hirshfeld Test Diff for C1 --C2 . 6.2 s.u.

PLAT232_ALERT_2_G Hirshfeld Test Diff (M-X) Sb1 --O1 . 6.1 s.u.

PLAT883_ALERT_1_G No Info/Value for _atom_sites_solution_primary . Please Do !

PLAT912_ALERT_4_G Missing # of FCF Reflections Above STh/L= 0.600 4 Note

PLAT933_ALERT_2_G Number of OMIT Records in Embedded .res File ... 4 Note

PLAT941_ALERT_3_G Average HKL Measurement Multiplicity .... 2.9 Low

PLAT965_ALERT_2_G The SHELXL WEIGHT Optimisation has not Converged Please Check

PLAT978_ALERT_2_G Number C-C Bonds with Positive Residual Density. 12 Info

---

PLATON version of 04/06/2020; check.def file version of 02/06/2020

Datablock Sb-Ph4-3PCO - ellipsoid plot

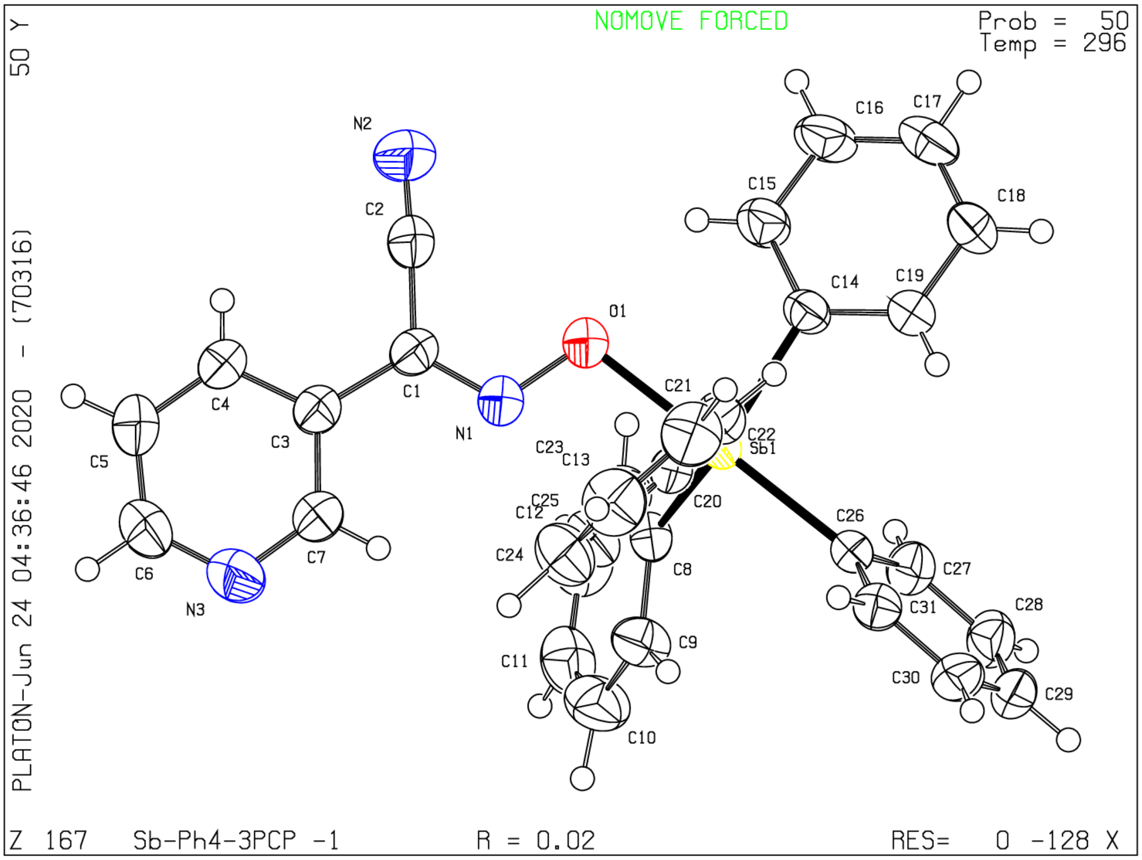

checkCIF/PLATON (basic structural check)

Structure factors have been supplied for datablock(s) Sb-Ph4-4PCO

No syntax errors found.
Please wait while processing ....

CIF dictionary
Interpreting this report

Structure factor report

Datablock: Sb-Ph4-4PCO

Bond precision: C-C = 0.0031 A Wavelength=0.71073

Cell:	a=15.3352(10)	b=9.7848(6)	c=19.6576(9)
	alpha=90	beta=119.272(4)	gamma=90

Temperature: 120 K

	Calculated	Reported
Volume	2573.0(3)	2573.0(3)
Space group	P 21/c	P 21/c
Hall group	-P 2ybc	-P 2ybc
Moiety formula	C31 H24 N3 O Sb	C31 H24 N3 O Sb
Sum formula	C31 H24 N3 O Sb	C31 H24 N3 O Sb
Mr	576.29	576.28
Dx,g cm-3	1.488	1.428
Z	4	4
Mu (mm-1)	1.101	1.099
F000	1160.0	1068.0
F000'	1158.02	
h,k,lmax	23,15,30	22,14,29
Nref	9749	9309
Tmin,Tmax	0.730,0.803	0.683,0.880
Tmin'	0.514	

Correction method= # Reported T Limits:

Tmin=0.683 Tmax=0.880 AbsCorr = NUMERICAL

Data completeness= 0.955 Theta(max)= 33.082

R(reflections)= 0.0286(7879)   wR2(reflections)= 0.0678(9309)

S = 1.098   Npar= 417

The following ALERTS were generated. Each ALERT has the format

test-name_ALERT_alert-type_alert-level.

Click on the hyperlinks for more details of the test.

Alert level C

DENSD01_ALERT_1_C The ratio of the submitted crystal density and that
calculated from the formula is outside the range 0.99 <> 1.01
Crystal density given = 1.428
Calculated crystal density = 1.488

PLAT046_ALERT_1_C Reported Z, MW and D(calc) are Inconsistent .... 1.488 Check

PLAT068_ALERT_1_C Reported F000 Differs from Calcd (or Missing)... Please Check

PLAT094_ALERT_2_C Ratio of Maximum / Minimum Residual Density .... 2.99 Report

PLAT971_ALERT_2_C Check Calcd Resid. Dens. 1.12A From C21 1.73 eA-3

Alert level G

PLAT128_ALERT_4_G Alternate Setting for Input Space Group P21/c P21/n Note

PLAT164_ALERT_4_G Nr. of Refined C-H H-Atoms in Heavy-Atom Struct. 23 Note

PLAT232_ALERT_2_G Hirshfeld Test Diff (M-X) Sb1 --O1 . 5.3 s.u.

PLAT883_ALERT_1_G No Info/Value for _atom_sites_solution_primary . Please Do !

PLAT912_ALERT_4_G Missing # of FCF Reflections Above STh/L= 0.600 400 Note

PLAT933_ALERT_2_G Number of OMIT Records in Embedded .res File ... 10 Note

PLAT941_ALERT_3_G Average HKL Measurement Multiplicity .... 4.5 Low

PLAT965_ALERT_2_G The SHELXL WEIGHT Optimisation has not Converged Please Check

PLAT978_ALERT_2_G Number C-C Bonds with Positive Residual Density. 15 Info

---

PLATON version of 04/06/2020; check.def file version of 02/06/2020

Datablock Sb-Ph4-4PCO - ellipsoid plot

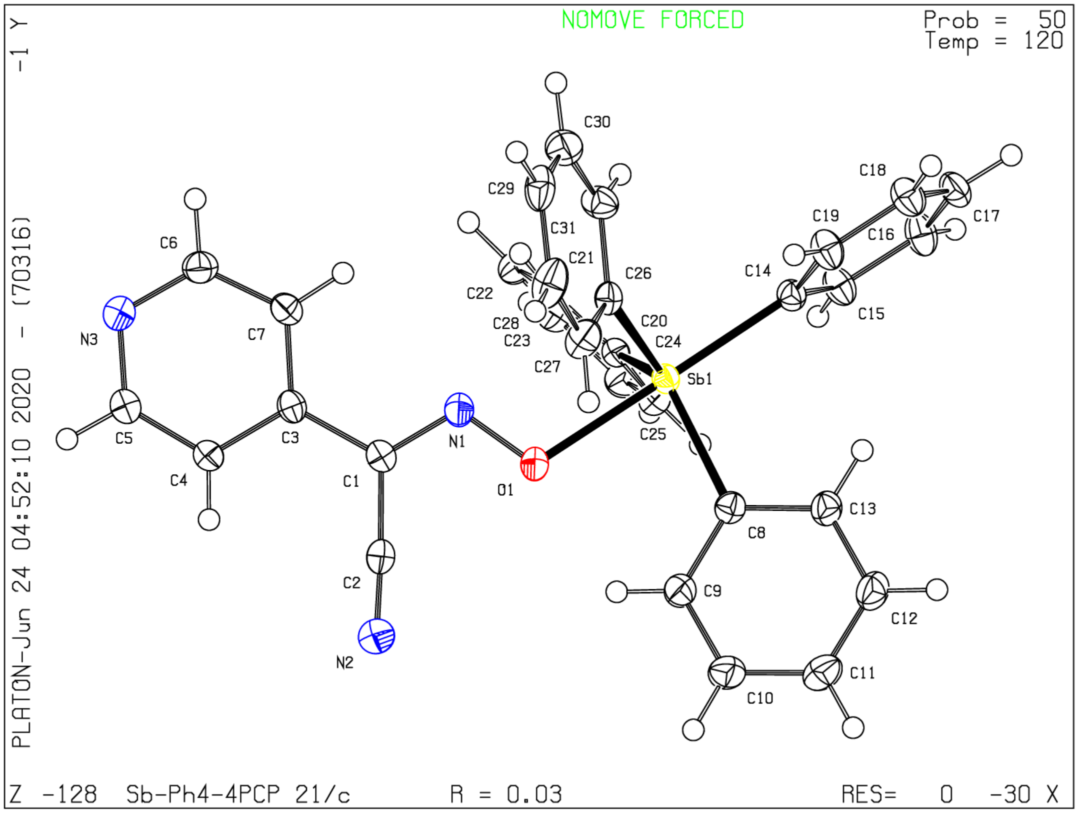

checkCIF/PLATON (basic structural check)

Structure factors have been supplied for datablock(s) Sb-pH4-ACO

No syntax errors found.
Please wait while processing ....

CIF dictionary
Interpreting this report

Structure factor report

Datablock: Sb-Ph4-ACO

Bond precision: C-C = 0.0034 A Wavelength=0.71073

Cell:	a=14.8336(8)	b=9.9060(6)	c=17.3977(10)
	alpha=90	beta=112.713(1)	gamma=90

Temperature: 120 K

	Calculated	Reported
Volume	2358.2(2)	2358.2(2)
Space group	P 21/c	P 21/c
Hall group	-P 2ybc	-P 2ybc
Moiety formula	C27 H22 N3 O2 Sb	C27 H22 N3 O2 Sb
Sum formula	C27 H22 N3 O2 Sb	C27 H22 N3 O2 Sb
Mr	542.24	542.22
Dx,g cm-3	1.527	1.527
Z	4	4
Mu (mm-1)	1.199	1.199
F000	1088.0	1088.0
F000'	1086.02	
h,k,lmax	22,15,26	22,15,26
Nref	8912	8465
Tmin,Tmax	0.864,0.901	0.648,0.953
Tmin'	0.839	

Correction method= # Reported T Limits:

Tmin=0.648 Tmax=0.953 AbsCorr = NUMERICAL

Data completeness= 0.950 Theta(max)= 33.019

R(reflections)= 0.0288(7070)   wR2(reflections)= 0.0726(8465)

S = 1.172   Npar= 386

The following ALERTS were generated. Each ALERT has the format

test-name_ALERT_alert-type_alert-level.

Click on the hyperlinks for more details of the test.

Alert level B

PLAT919_ALERT_3_B Reflection # Likely Affected by the Beamstop ... 1 Check

PLAT934_ALERT_3_B Number of (Iobs-Icalc)/Sigma(W) > 10 Outliers .. 8 Check

---

Alert level C

PLAT094_ALERT_2_C Ratio of Maximum / Minimum Residual Density .... 2.71 Report

PLAT420_ALERT_2_C D-H Without Acceptor N3 --H2N3 . Please Check

PLAT971_ALERT_2_C Check Calcd Resid. Dens. 2.26A From C5 1.62 eA-3

Alert level G

PLAT164_ALERT_4_G Nr. of Refined C-H H-Atoms in Heavy-Atom Struct. 20 Note

PLAT720_ALERT_4_G Number of Unusual/Non-Standard Labels .... 2 Note

PLAT883_ALERT_1_G No Info/Value for _atom_sites_solution_primary . Please Do !

PLAT912_ALERT_4_G Missing # of FCF Reflections Above STh/L= 0.600 447 Note

PLAT941_ALERT_3_G Average HKL Measurement Multiplicity .... 4.5 Low

PLAT965_ALERT_2_G The SHELXL WEIGHT Optimisation has not Converged Please Check

PLAT978_ALERT_2_G Number C-C Bonds with Positive Residual Density. 4 Info

---

PLATON version of 04/06/2020; check.def file version of 02/06/2020

Datablock Sb-Ph4-ACO - ellipsoid plot

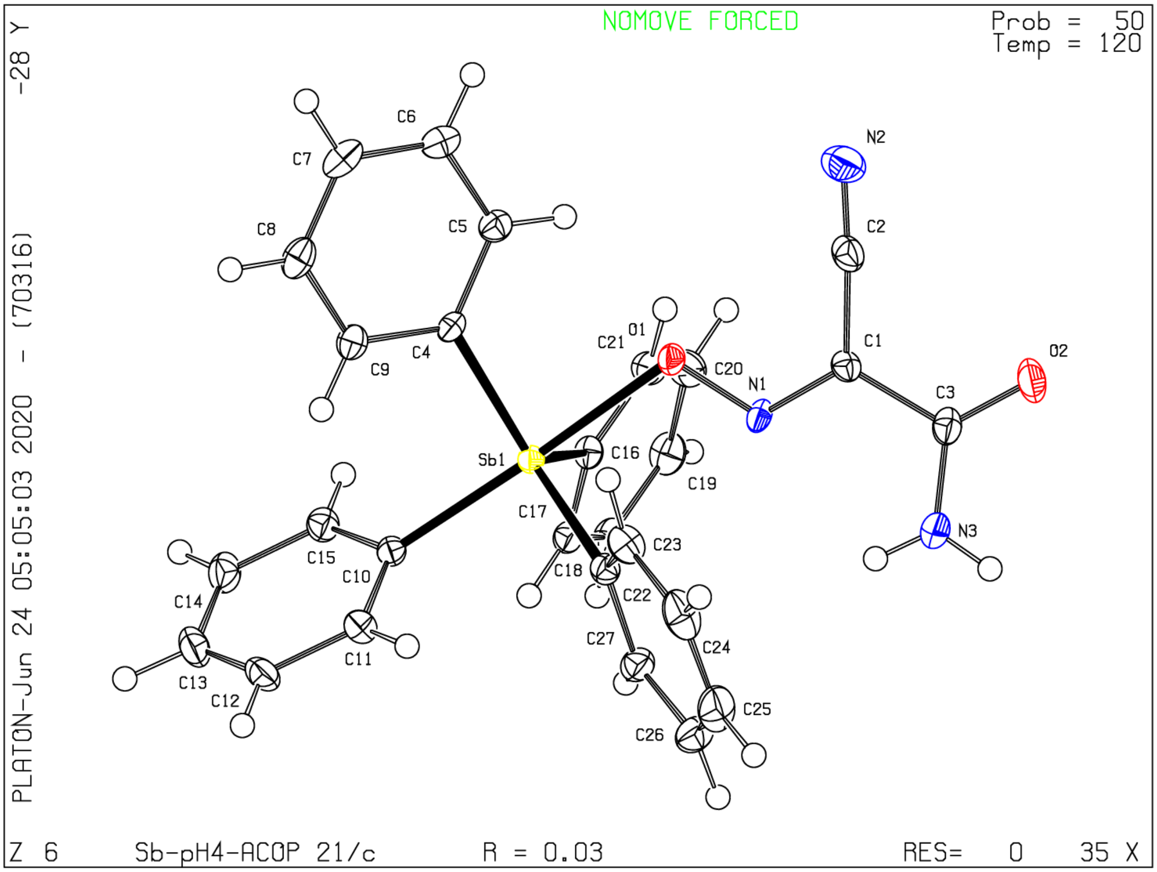

checkCIF/PLATON (basic structural check)

Structure factors have been supplied for datablock(s) Sb-Ph4-ECI-oxo-dimer

No syntax errors found.
Please wait while processing ....

CIF dictionary
Interpreting this report

Structure factor report

Datablock: Sb-Ph4-ECO-oxo-dimer

Bond precision: C-C = 0.0024 A Wavelength=0.71073

Cell:	a=9.1828(9)	b=10.0829(10)	c=13.4714(16)
	alpha=111.957(1)	beta=103.200(2)	gamma=95.405(1)

Temperature: 120 K

	Calculated	Reported
Volume	1103.8(2)	1103.8(2)
Space group	P -1	P -1
Hall group	-P 1	-P 1
Moiety formula	C46 H40 N4 O7 Sb2	C46 H40 N4 O7 Sb2
Sum formula	C46 H40 N4 O7 Sb2	C46 H40 N4 O7 Sb2
Mr	1004.34	1004.32
Dx,g cm-3	1.511	1.511
Z	1	1
Mu (mm-1)	1.277	1.277
F000	502.0	502.0
F000'	501.02	
h,k,lmax	13,14,19	13,14,19
Nref	6741	6688
Tmin,Tmax	0.712,0.795	0.824,1.000
Tmin'	0.282	

Correction method= # Reported T Limits:

Tmin=0.824 Tmax=1.000 AbsCorr = NUMERICAL

Data completeness= 0.992 Theta(max)= 30.508

R(reflections)= 0.0188(6300) wR2(reflections)= 0.0447(6688)

S = 1.079 Npar= 348

The following ALERTS were generated. Each ALERT has the format

test-name_ALERT_alert-type_alert-level.

Click on the hyperlinks for more details of the test.

Alert level C

PLAT094_ALERT_2_C Ratio of Maximum / Minimum Residual Density .... 2.52 Report

---

Alert level G

PLAT063_ALERT_4_G Crystal Size Possibly too Large for Beam Size .. 0.98 mm

PLAT164_ALERT_4_G Nr. of Refined C-H H-Atoms in Heavy-Atom Struct. 20 Note

PLAT720_ALERT_4_G Number of Unusual/Non-Standard Labels .... 1 Note

PLAT883_ALERT_1_G No Info/Value for _atom_sites_solution_primary . Please Do !

PLAT912_ALERT_4_G Missing # of FCF Reflections Above STh/L= 0.600 52 Note

PLAT941_ALERT_3_G Average HKL Measurement Multiplicity .... 2.6 Low

PLAT965_ALERT_2_G The SHELXL WEIGHT Optimisation has not Converged Please Check

PLAT978_ALERT_2_G Number C-C Bonds with Positive Residual Density. 16 Info

---

PLATON version of 04/06/2020; check.def file version of 02/06/2020

Datablock Sb-Ph4-ECO-oxo-dimer - ellipsoid plot

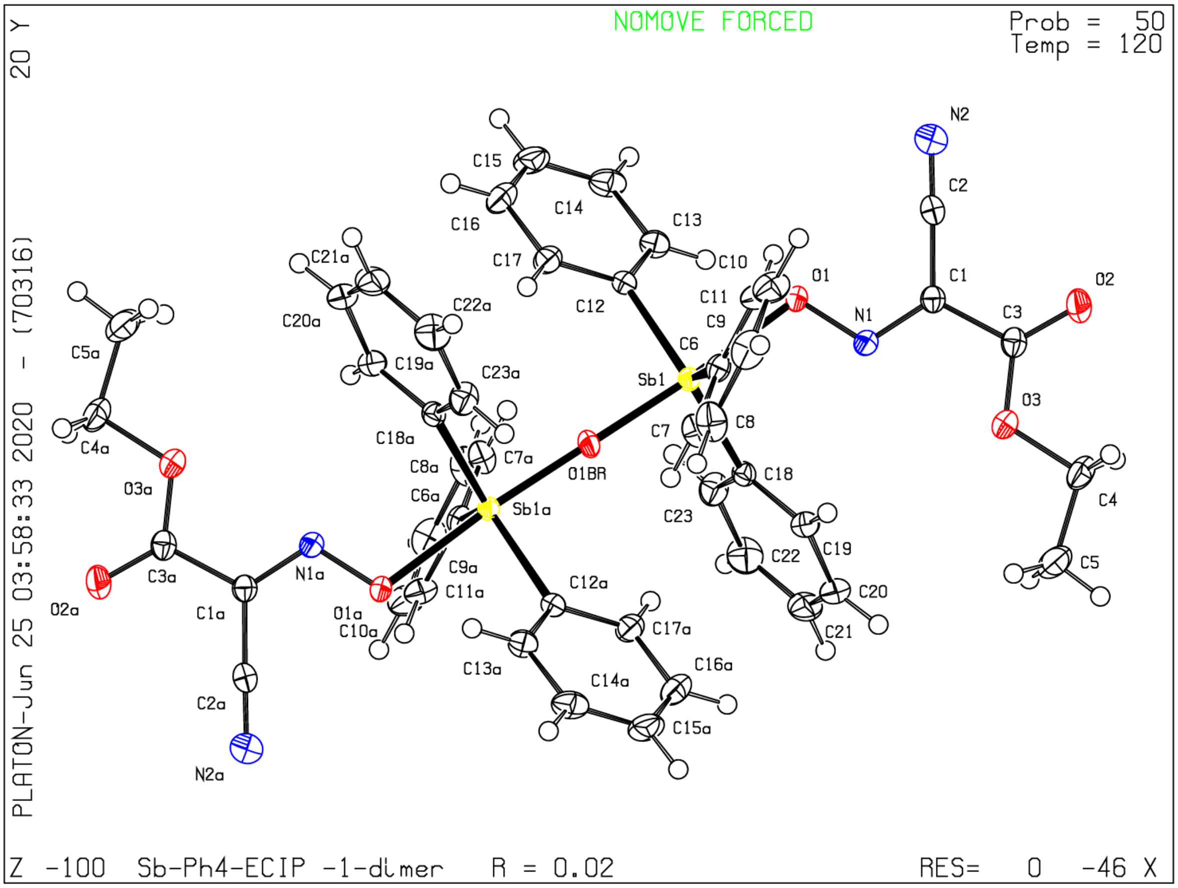
NOMOVE FORCED
Prob = 50
Temp = 120
20 Y
PLATON-Jun 25 03:58:33 2020 - (70316)
Z -100 Sb-Ph4-ECIP -1-dimer R = 0.02
RES= 0 -46 X
N2
C2
C1
C3
O2
O3
C4
C5
N1
O1
Sb1
O1BR
Sb1a
C6
C7
C8
C9
C10
C11
C12
C13
C14
C15
C16
C17
C18
C19
C20
C21
C22
C23
C1a
C2a
N2a
C3a
O2a
O3a
C4a
C5a
N1a
O1a
C6a
C7a
C8a
C9a
C10a
C11a
C12a
C13a
C14a
C15a
C16a
C17a
C18a
C19a
C20a
C21a
C22a
C23a

checkCIF/PLATON (basic structural check)

Structure factors have been supplied for datablock(s) Sb-Ph4-ECO-monomer

No syntax errors found.
Please wait while processing ....

CIF dictionary
Interpreting this report

Structure factor report

Datablock: Sb-Ph4-ECO-monomer

Bond precision: C-C = 0.0080 A   Wavelength=0.71073

Cell: a=9.7871(6) b=14.9208(9) c=17.6984(11)

alpha=90 beta=94.613(1) gamma=90

Temperature: 120 K

	Calculated	Reported
Volume	2576.2(3)	2576.1(3)
Space group	P n	P n
Hall group	P -2yac	P -2yac
Moiety formula	C29 H25 N2 O3 Sb	C29 H25 N2 O3 Sb
Sum formula	C29 H25 N2 O3 Sb	C29 H25 N2 O3 Sb
Mr	571.27	571.26
Dx,g cm-3	1.473	1.473
Z	4	4
Mu (mm-1)	1.103	1.103
F000	1152.0	1152.0
F000'	1150.07	
h,k,lmax	15,22,27	14,22,26
Nref	19496[9759]	9092
Tmin,Tmax	0.815,0.849	0.653,0.747
Tmin'	0.791	

Correction method= # Reported T Limits:

Tmin=0.653 Tmax=0.747 AbsCorr = MULTI-SCAN

Data completeness= 0.93/0.47   Theta(max)= 33.076

R(reflections)= 0.0351(8454)   wR2(reflections)= 0.0774(9092)

S = 1.193   Npar= 654

The following ALERTS were generated. Each ALERT has the format

test-name_ALERT_alert-type_alert-level.

Click on the hyperlinks for more details of the test.

Alert level C

STRVA01_ALERT_4_C Flack test results are ambiguous.

From the CIF: _refine_ls_abs_structure_Flack 0.530

From the CIF: _refine_ls_abs_structure_Flack_su 0.040

PLAT220_ALERT_2_C NonSolvent Resd 1 C Ueq(max) / Ueq(min) Range 3.2 Ratio

PLAT220_ALERT_2_C NonSolvent Resd 2 C Ueq(max) / Ueq(min) Range 3.3 Ratio

PLAT222_ALERT_3_C NonSolvent Resd 1 H Uiso(max)/Uiso(min) Range 6.6 Ratio

PLAT342_ALERT_3_C Low Bond Precision on C-C Bonds .... 0.00802 Ang.

PLAT907_ALERT_2_C Flack x > 0.5, Structure Needs to be Inverted? . 0.53 Check

PLAT914_ALERT_3_C No Bijvoet Pairs in FCF for Non-centro Structure Please Check

PLAT971_ALERT_2_C Check Calcd Resid. Dens. 0.85A From Sb1B 1.73 eA-3

And 2 other PLAT971 Alerts

PLAT971_ALERT_2_C Check Calcd Resid. Dens. 1.30A From C18B 1.51 eA-3

PLAT971_ALERT_2_C Check Calcd Resid. Dens. 0.64A From Sb1A 1.51 eA-3

PLAT977_ALERT_2_C Check Negative Difference Density on H13A -0.41 eA-3

And 2 other PLAT977 Alerts

PLAT977_ALERT_2_C Check Negative Difference Density on H16B -0.32 eA-3

PLAT977_ALERT_2_C Check Negative Difference Density on H26A -0.35 eA-3

Alert level G

```
PLAT083_ALERT_2_G SHELXL Second Parameter in WGHT  Unusually Large       5.57 Why ?
PLAT164_ALERT_4_G Nr. of Refined C-H H-Atoms in Heavy-Atom Struct.          5 Note
PLAT720_ALERT_4_G Number of Unusual/Non-Standard Labels ..........         10 Note
PLAT883_ALERT_1_G No Info/Value for _atom_sites_solution_primary .     Please Do !
PLAT912_ALERT_4_G Missing # of FCF Reflections Above STh/L=  0.600        656 Note
PLAT933_ALERT_2_G Number of OMIT Records in Embedded .res File ...          5 Note
PLAT941_ALERT_3_G Average HKL Measurement Multiplicity ...........        1.0 Low
PLAT961_ALERT_5_G Dataset Contains no Negative Intensities .......     Please Check
PLAT978_ALERT_2_G Number C-C Bonds with Positive Residual Density.          0 Info
PLAT992_ALERT_5_G Repd & Actual _reflns_number_gt Values Differ by          8 Check
```

PLATON version of 04/06/2020; check.def file version of 02/06/2020

Datablock Sb-Ph4-ECO-monomer - ellipsoid plot

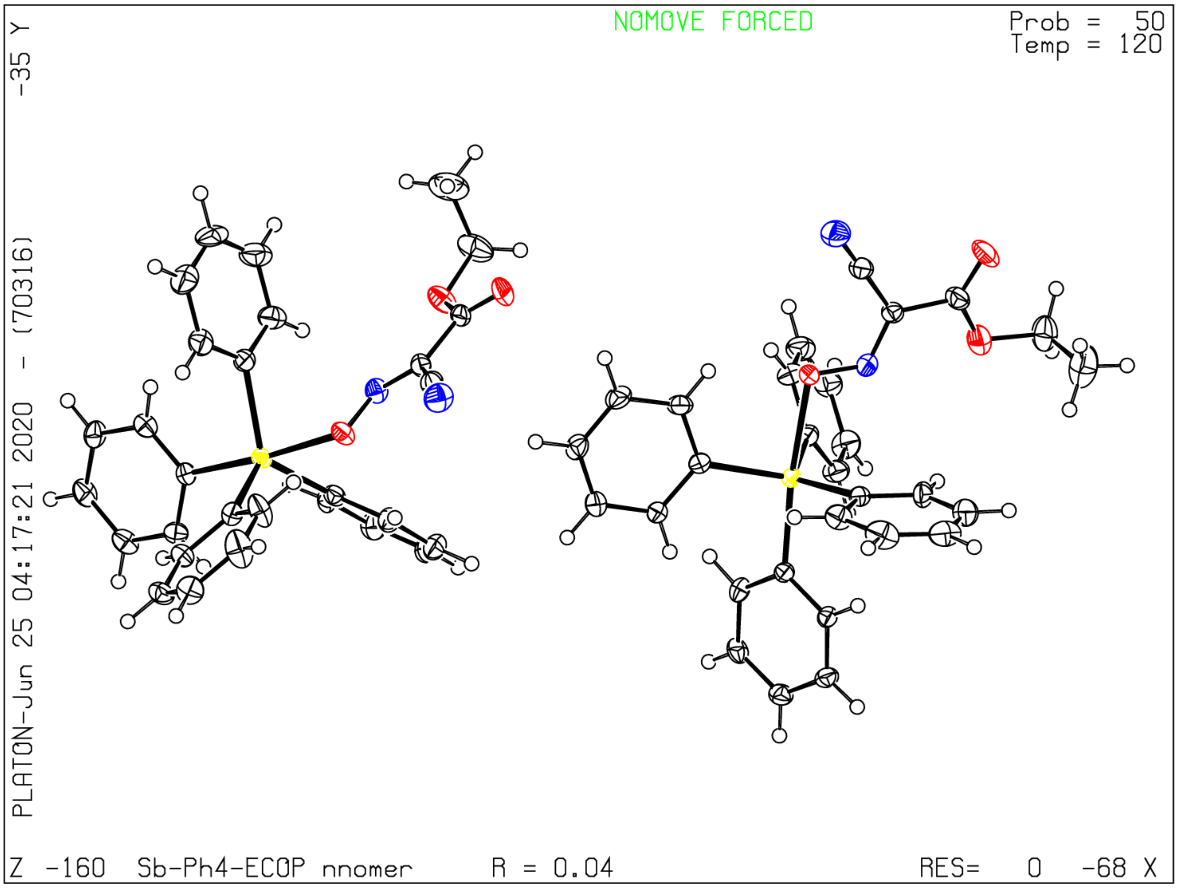
NOMOVE FORCED
Prob = 50
Temp = 120
-35 Y
PLATON-Jun 25 04:17:21 2020 - (70316)
Z -160 Sb-Ph4-ECOP nnomer
R = 0.04
RES= 0 -68 X

checkCIF/PLATON (basic structural check)

Structure factors have been supplied for datablock(s) Sb-Ph4-MCO

No syntax errors found.
Please wait while processing ....

CIF dictionary
Interpreting this report

Structure factor report

Datablock: Sb-Ph4-MCO

Bond precision: C-C = 0.0065 A   Wavelength=0.71073

Cell:	a=17.6451(4)	b=10.8777(3)	c=28.3876(7)
	alpha=90	beta=94.836(1)	gamma=90
Temperature:	100 K		

	Calculated	Reported
Volume	5429.3(2)	5429.3(2)
Space group	P 21/c	P 21/c
Hall group	-P 2ybc	-P 2ybc
Moiety formula	C31 H28 N3 O3 Sb	C31 H28 N3 O3 Sb
Sum formula	C31 H28 N3 O3 Sb	C31 H28 N3 O3 Sb
Mr	612.32	612.31
Dx,g cm-3	1.498	1.498
Z	8	8
Mu (mm-1)	1.054	1.054
F000	2480.0	2480.0
F000'	2476.19	
h,k,lmax	22,13,35	22,13,35
Nref	11273	11251
Tmin,Tmax	0.863,0.881	0.689,0.745

Tmin' 0.863

Correction method= # Reported T Limits:
Tmin=0.689 Tmax=0.745 AbsCorr = MULTI-SCAN

Data completeness= 0.998 Theta(max)= 26.516

R(reflections)= 0.0447(9235) wR2(reflections)= 0.1109(11251)

S = 1.114 Npar= 750

The following ALERTS were generated. Each ALERT has the format

test-name_ALERT_alert-type_alert-level.

Click on the hyperlinks for more details of the test.

Alert level C

PLAT094_ALERT_2_C Ratio of Maximum / Minimum Residual Density .... 3.19 Report

PLAT222_ALERT_3_C NonSolvent Resd 2 H Uiso(max)/Uiso(min) Range 7.7 Ratio

PLAT245_ALERT_2_C U(iso) H28A Smaller than U(eq) C28A by 0.017 Ang**2

And 3 other PLAT245 Alerts

PLAT245_ALERT_2_C U(iso) H13B Smaller than U(eq) C13B by 0.012 Ang**2

PLAT245_ALERT_2_C U(iso) H21B Smaller than U(eq) C21B by 0.014 Ang**2

PLAT245_ALERT_2_C U(iso) H30B Smaller than U(eq) C30B by 0.012 Ang**2

PLAT906_ALERT_3_C Large K Value in the Analysis of Variance ...... 4.570 Check

PLAT911_ALERT_3_C Missing FCF Refl Between Thmin & STh/L= 0.600 9 Report

PLAT971_ALERT_2_C Check Calcd Resid. Dens. 1.05A From C26B 1.94 eA-3

PLAT971_ALERT_2_C Check Calcd Resid. Dens. 1.18A From Sb1A 1.65 eA-3

PLAT975_ALERT_2_C Check Calcd Resid. Dens. 0.81A From O1A 0.57 eA-3

Alert level G

PLAT083_ALERT_2_G SHELXL Second Parameter in WGHT Unusually Large 14.34 Why ?

PLAT164_ALERT_4_G Nr. of Refined C-H H-Atoms in Heavy-Atom Struct. 16 Note

PLAT232_ALERT_2_G Hirshfeld Test Diff (M-X) Sb1A --O1A . 5.3 s.u.

PLAT398_ALERT_2_G Deviating C-O-C Angle From 120 for O3B 109.9 Degree

PLAT720_ALERT_4_G Number of Unusual/Non-Standard Labels .... 16 Note

PLAT883_ALERT_1_G No Info/Value for _atom_sites_solution_primary . Please Do !

PLAT912_ALERT_4_G Missing # of FCF Reflections Above STh/L= 0.600 15 Note

PLAT933_ALERT_2_G Number of OMIT Records in Embedded .res File ... 10 Note

PLAT965_ALERT_2_G The SHELXL WEIGHT Optimisation has not Converged Please Check

PLAT978_ALERT_2_G Number C-C Bonds with Positive Residual Density. 2 Info

---

PLATON version of 04/06/2020; check.def file version of 02/06/2020

Datablock Sb-Ph4-MCO - ellipsoid plot

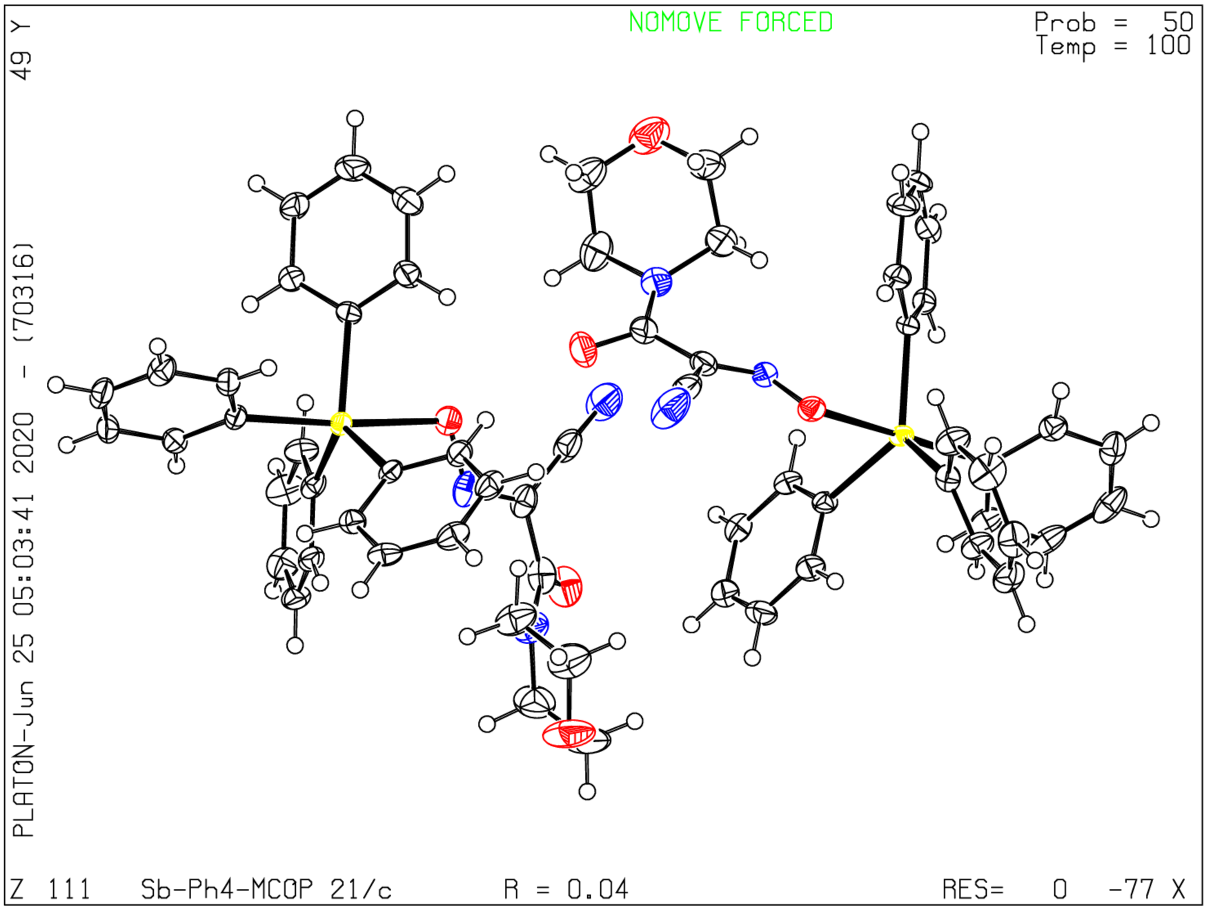
NOMOVE FORCED
Prob = 50
Temp = 100
49 Y
PLATON-Jun 25 05:03:41 2020 - (70316)
Z 111
Sb-Ph4-MCOP 21/c
R = 0.04
RES= 0 -77 X
